# Gut microbiota and metabolic profiles in chronic intermittent hypoxia-induced rats: disease-associated dysbiosis and metabolic disturbances

**DOI:** 10.3389/fendo.2023.1224396

**Published:** 2024-01-12

**Authors:** Cong Li, Song Shi

**Affiliations:** Department of Otorhinolaryngology, Tongren Hospital, Shanghai Jiao Tong University School of Medicine, Shanghai, China

**Keywords:** obstructive sleep apnea, chronic intermittent hypoxia, gut microbiota, metabolomics, cAMP pathway

## Abstract

**Aim:**

Chronic intermittent hypoxia (CIH) is a key characteristic of obstructive sleep apnea (OSA) syndrome, a chronic respiratory disorder. The mechanisms of CIH-induced metabolic disturbance and histopathological damage remain unclear.

**Methods:**

CIH-induced rats underwent daily 8-h CIH, characterized by oxygen levels decreasing from 21% to 8.5% over 4 min, remaining for 2 min, and quickly returning to 21% for 1 min. The control rats received a continuous 21% oxygen supply. The levels of hypersensitive C reactive protein (h-CRP), tumor necrosis factor-α (TNF-α), interleukin 6 (IL-6), interleukin 8 (IL-8), and nuclear factor kappa-B (NF-κB) were measured by ELISA. Histological analysis of the soft palates was conducted using HE staining. The microbial profiling of fecal samples was carried out by Accu16STM assay. Untargeted metabolomics of serum and soft palate tissue samples were analyzed by UPLC-MS. The protein expression of cAMP-related pathways in the soft palate was determined by Western blot.

**Results:**

After 28 h of CIH induction, a significant increase in pro-inflammatory cytokines was observed in the serum, along with mucosal layer thickening and soft palate tissue hypertrophy. CIH induction altered the diversity and composition of fecal microbiota, specifically reducing beneficial bacteria while increasing harmful bacteria/opportunistic pathogens. Notably, CIH induction led to a significant enrichment of genera such as *Dorea*, *Oscillibacter*, *Enteractinococcus*, *Paenibacillus*, *Globicatella*, and *Flaviflexus* genera. Meanwhile, Additionally, CIH induction had a notable impact on 108 serum marker metabolites. These marker metabolites, primarily involving amino acids, organic acids, and a limited number of flavonoids or sterols, were associated with protein transport, digestion and absorption, amino acid synthesis and metabolism, as well as cancer development. Furthermore, these differential serum metabolites significantly affected 175 differential metabolites in soft palate tissue, mainly related to cancer development, signaling pathways, amino acid metabolism, nucleotide precursor or intermediate metabolism, respiratory processes, and disease. Importantly, CIH induction could significantly affect the expression of the cAMP pathway in soft palate tissue.

**Conclusions:**

Our findings suggest that targeting differential metabolites in serum and soft palate tissue may represent a new approach to clinical intervention and treatment of OSA simulated by the CIH.

## 1 Introduction

Obstructive sleep apnea (OSA) is a chronic respiratory disorder that affects up to 50% of the population, depending on the country, and is prevalent among nearly one billion adults aged 30 to 69 (1). It is characterized by recurrent upper airway obstruction and reduced or even stopped airflow during sleep, and this reduced airflow may lead to intermittent hypoxia and sleep disturbance (2, 3). Current studies have shown that chronic intermittent hypoxia (CIH) is the most critical pathological feature of OSA, which will lead to repeated hypoxia-reperfusion in tissues and organs, cause inflammation and oxidative stress, and eventually induce organ tissue damage (4). Long-term OSA is closely related to the occurrence and development of Alzheimer’s disease (5), coronary atherosclerotic heart disease (6), metabolic disease (7), and cognitive dysfunction (8). Given the OSA-induced damage to various organs and tissues, early diagnosis and intervention are crucial for preventing the further development of OSA. At present, overnight polysomnography is still the gold standard for clinical diagnosis of OSA (9), but due to its high price, long waiting time, discomfort, and induced sleep disturbance, it is still difficult to clinically use it for extensive screening and early diagnosis for OSA patients. In addition to the screening and diagnosis for OSA, the most effective clinical treatment for OSA patients is continuous positive airway pressure (CPAP) (10). Although continuous CPAP therapy for several months can significantly improve their cognitive impairment, the impaired cognitive function has not yet been fully recovered (11). Therefore, finding easy-detected biomarkers with high sensitivity and specificity and key targets for CIH induction is crucial for the prevention and treatment of OSA.

The human gut microbiota is a sophisticated community of microorganisms, and establishing a mutually beneficial relationship with them is crucial for maintaining human health (12). Gut microbiota dysbiosis is widely recognized as a crucial contributor to the pathogenesis of OSA-related diseases. OSA can perturb the gut microbiome and lead to dysbiosis. Specifically, OSA-induced apnea and hypopnea trigger an elevated abundance of anaerobic and facultative anaerobic bacteria and a reduced abundance of obligate aerobic bacteria in the gut microbiota (13). In addition, a causal relationship between gut microbiota and OSA-induced hypertension has been demonstrated by Durgan et al., with an increase in the abundance of the lactate-producing family *Coriobacteriaceae* and a decrease in the butyrate-producing genus *Eubacterium* (14). Similarly, Ko et al. reported that patients with OSA-hypopnea syndrome exhibit gut microbiota dysbiosis characterized by a decrease in short-chain fatty acid (SCFA)-producing bacteria and an increase in pathogenic bacteria, with the *Ruminococcus* enterotype posing the highest risk for OSA patients (15). Long-term OSA can disrupt the diversity and stability of the gut microbiota, leading to a decrease in the abundance of commensal bacteria and an increase in opportunistic pathogens. This process can trigger intestinal inflammation and oxidative stress, ultimately culminating in OSA-associated systemic hypertension, coronary artery disease, obesity, and diabetes mellitus (16). Therefore, modulation of the gut microbiota represents a promising strategy for OSA treatment. Despite the demonstration of dysbiosis in OSA hosts, the underlying mechanisms by which OSA induces changes in the gut microbiota remain incompletely understood.

Metabolomics, a subfield of omics, involves the comprehensive identification and quantification of small-molecule metabolites (with molecular weight <1500 Da) and is widely recognized for its high sensitivity, resolution, and throughput (17). Untargeted metabolomics has proven particularly useful in providing a global overview of endogenous metabolic fluctuations triggered by diseases. Consequently, it has been extensively employed in the identification of metabolite biomarkers for OSA diagnosis and prognosis. For instance, Engeli et al. (18) reported significantly higher levels of endocannabinoids in the plasma of OSA patients compared to obese individuals without OSA. Among these endocannabinoids, anandamide exhibited greater effects on blood pressure in OSA patients than obesity, insulin resistance, and inflammation, thereby serving as a potential marker for cardiovascular event risk prediction in OSA patients. Similarly, Ezzedini et al. (19) demonstrated that children with OSA had lower levels of palmitoleic acid and oleic acid but higher levels of stearic acid in tonsil tissue relative to children with chronic tonsillitis. Moreover, they identified a positive correlation between the content of oleic acid in tonsil tissue and body mass index, snoring, and tonsil hypertrophy. These findings provide a compelling rationale for implementing further metabolomic analyses to identify OSA-related biomarkers.

In this study, CIH induction was used to simulate OSA in rats, and the typical characteristics of rats after CIH induction were analyzed. Feces were collected from rats to analyze their gut microbial composition, followed by serum and soft palate tissue metabolic profiles associated with CIH. Key KEGG-enriched pathways in tissue metabolic profiles were selected for further expression validation to explore possible mechanisms of CIH-simulated OSA.

Compared with previous related research reports, the microbiome profiling of CIH-simulated OSA rats was analyzed innovatively using absolute quantitative analysis technology. Furthermore, an integrated metabolomic analysis of both serum and soft palate tissue revealed typical differential metabolites that could reflect the characteristics of OSA, and the cyclic adenosine monophosphate (cAMP) pathway might be a key target for differences in soft palate histopathology.

## 2 Materials and methods

### 2.1 Animal treatments

All experimental procedures were conducted in strict accordance with the Institutional Animal Use and Care Committee guidelines of the Second Military Medical University (approval number 2022-010). Six-week-old male Sprague-Dawley rats (170 ± 2 g) were randomly assigned to two groups, each consisting of 5 rats, and housed in polycarbonate cages. Rats were provided with standard food and water and maintained on a 12-h light/dark cycle. The CIH group rats were exposed to intermittent hypoxia using plastic cages equipped with intermittent hypoxia devices for 8 h per day, with CIH being performed during the 12-h light cycle to align with the animals’ sleep cycle. CIH rats were subjected to CIH for 28 consecutive days in 8-h daily cycles from 9:00 a.m. to 5:00 p.m. During each cycle, the internal oxygen concentration was reduced from 21% to 8.5% over 4 min and maintained at this level for 2 min. The oxygen concentration was then rapidly restored to 21% for 2 min and maintained at this level for 1 min. Animals in the control group were housed in the same chamber but were provided with a constant flow of 21% oxygen. At the end of the experimental period, blood samples were collected from the rats via orbital bleeding, and the rats were subsequently euthanized. The soft palate tissues were swiftly harvested on ice, rinsed with deionized water, and dried using filter paper. These tissues were then promptly stored at -80°C. Simultaneously, sterile collection of rat colon feces was carried out and stored at -80°C.

### 2.2 Biochemical parameters

Partial blood samples from the rats were collected into blood collection tubes. Within 30 min, the samples were centrifuged at 4°C and 3000 rpm for 15 min. The obtained rat serum samples (300 μL) were stored at -80°C. Commercially available assay kits (Thermo Fisher Scientific, Waltham, MA, USA) were used to measure the levels of various biomarkers in the serum, including hypersensitive C reactive protein (h-CRP), tumor necrosis factor-α (TNF-α), interleukin 6 (IL-6), interleukin 8 (IL-8), and nuclear factor kappa-B (NF-κB). Briefly, 50 μL of diluted samples (diluted 5 times) were added to antibody-coated microplates, followed by the addition of 100 μL of enzyme-labeling reagents. The plates were then incubated at 37°C for 60 min. After washing, chromogenic substrates were added, and the plates were incubated at 37°C in the dark for 15 min. The reaction was terminated, and the absorbance values for each well were determined.

### 2.3 Histology analysis

Soft palate tissues from both groups of rats underwent a series of processing steps. They were dehydrated, fixed with formalin, and later embedded in paraffin. Following this, the paraffin-embedded tissues were cut into sections after drying at 60°C for 30-60 min. These tissue sections were then subjected to transparent using xylene for 15 min, followed by treatment with various ethanol concentrations. Subsequently, Hematoxylin and Eosin (H&E) staining to assess the histological alterations in the soft palate mucosa and muscles was conducted.

### 2.4 Microbiome profiling for absolute quantification of 16S rRNA amplicon sequencing

The Accu16STM (Accurate 16S absolute quantification sequencing) assay was performed by Genesky Biotechnologies Inc. (Shanghai, China). Briefly, genomic DNA was extracted from cecal contents using the FastDNA SPIN Kit (MP Biomedicals, San-ta Ana, CA) according to the manufacturer’s instructions. The integrity, concentration, and purity of the genomic DNA were assessed using agarose gel electrophoresis, Nanodrop 2000 (ThermoFisher Scientific, Waltham, MA, USA), and Qubit3.0 Spectro-photometer (ThermoFisher Scientific, Waltham, MA, USA), respectively. To enable absolute quantification, spike-ins with conserved regions similar to natural 16S rRNA genes but with variable regions replaced by random sequences (~40% GC content) were artificially synthesized. A mixture of spike-ins with known gradient copy numbers was added to the sample DNA. The V3-V4 hypervariable regions of the 16S rRNA gene and spike-ins were amplified using primers 341F (5-CCTACGGGNGGCWGCAG-3) and 805R (5-GACTACHVGGGTATCTAATCC-3), respectively. Subsequently, the amplified products were sequenced using the Illumina NovaSeq 6000 sequencer (San Diego, CA, USA).5.4.2 Illumina read data processing and analysis.

The raw read sequences were processed using QIIME2. The cutadapt plugin was used to trim adaptor and primer sequences. Quality control and identification of amplicon sequence variants (ASVs) were performed using the DADA2 plugin. Taxonomic assignments of ASV representative sequences were conducted with a confidence threshold of 0.8 using a pre-trained Naive Bayes classifier trained on the RDP (version 11.5). Next, the spike-in sequences were identified, and reads were counted. A standard curve was generated for each sample, correlating read counts with spike-in copy numbers. The absolute copy number of each ASV in each sample was calculated based on the read counts of the corresponding ASV. Since the spike-in sequence was not part of the sample’s native microbiota, it was removed in subsequent analyses to focus on the analysis of the sample’s microbiota.

### 2.5 Untargeted metabolomics

Serum and soft palate tissue samples were analyzed using a Thermo Q Exactive mass spectrometer coupled with a Thermo Vanquish UPLC system (Thermo Fisher Scientific, Waltham, MA, USA). Chromatographic separation was performed on an ACQUITY UPLC^®^ HSS T3 column (2.1 mm × 150 mm, 1.8 μm; Waters, Milford, MA, USA) at a temperature of 40°C. For positive-ion mode elution, a mobile phase consisting of 0.1% formic acid in acetonitrile (C) and 0.1% formic acid in water (D) was used at a flow rate of 0.25 mL/min. In negative-ion mode, the mobile phase consisted of acetonitrile (A) and 5 mM ammonium formate in water (B) at the same flow rate. The gradient elution program was as follows: (1) for positive mode: 0-1 min, 2% C; 1-9 min, 2%-50% C; 9-12 min, 50%-98% C; 12-13.5 min, 98% C; 13.5-14 min, 98%-2% C; 14-20 min, 2% C; (2) for negative mode: 0-1 min, 2% A; 1-9 min, 2%-50% A; 9-12 min, 50%-98% A; 12-13.5 min, 98% A; 13.5-14 min, 98%-2% A; 14-17 min, 2% A.

The mass spectrometry analysis was performed using a high-resolution Thermo Q Exactive mass spectrometer. The primary full scan was conducted at a resolution of 70000, scanning the range of m/z 81-1000. High-energy collision dissociation (HCD) with a collision voltage of 30% was used for secondary fragmentation. The secondary resolution was set to 17500, and the top 10 samples were selected for collection. Dynamic exclusion was implemented to avoid redundant information. Electrospray ionization (ESI) in both positive-ion and negative-ion modes was used, with a spray voltage of 3.50 kV for the positive mode and 2.50 kV for the negative mode. Sheath gas and auxiliary gas were employed to aid the ionization process, and the capillary temperature was set to 325°C.

### 2.6 Western blotting assay

Western blotting analysis was carried out as reported previously by our lab (20). Briefly, after the total protein of soft palate tissues was extracted and separated, the samples were transferred onto a polyvinylidene fluoride (PVDF) membrane. Following washing, the membrane was incubated overnight at 4°C with primary antibodies against cAMP (1:1000, Abcam, ab76238), protein kinase A (PKA) (1:1000, Abcam, ab75991), cAMP-regulated guanine nucleotide exchange factor II (Epac2) (1:1000, Abcam, ab193665), rat sarcoma protein (Ras) (1:1000, Abcam, ab52939), c-Jun N-terminal kinase 1/2 (JNK1/2) (1:1000, Abcam, ab4821), Annexiv V (1:500, Abcam, ab14196), GAPDH (1:1000, Abcam, ab8245), and Tubulin (1:1000, Abcam, ab44928). After washing, the membrane was incubated with a horseradish peroxidase (HRP)-conjugated secondary antibody for 30 min at room temperature, followed by color development using a mixed solution. The image analyzer quantitative system was used to quantitatively determine the intensity of the target and reference proteins.

### 2.7 Statistical analysis

The statistical data were presented as the mean ± standard deviation. Group comparisons were performed by Student’s t-test, and p < 0.05 was applied for statistical significance. In sequencing data, inter-group microbial diversity indices in colon fecal samples were assessed using the Wilcoxon rank-sum test (p < 0.05 for significance). The Bonferroni method to correct p values for multiple hypothesis testing was applied to evaluate whether there were significant differences in species diversity between groups. Wilcoxon rank-sum tests were employed to compare the bacterial abundances at the phylum and genus levels between the two groups, with p < 0.05 as the threshold for significant differences. Pearson correlation analysis was used to calculate Pearson correlation coefficients between species, and species with p < 0.05 were selected to construct a network of dominant species associations. Non-parametric Kruskal-Wallis rank-sum tests were utilized to screen for species with significantly different relative abundances between different groups. Subsequently, paired Wilcoxon rank-sum tests were conducted for inter-subgroup differential analysis. Lastly, linear discriminant analysis (LDA) was used to evaluate the effect values of significantly different species (|LDA| > 2, p < 0.05). Differences in KEGG abundance between the two groups were compared using Wilcoxon analysis (p < 0.05 for significance). *, p < 0.05; **, p < 0.01; ***, p < 0.001; ****, p < 0.0001; ns, not significant.

## 3 Results

### 3.1 Effect of CIH induction on serum pro-inflammatory cytokines and soft palate tissue

**Figure 1:**
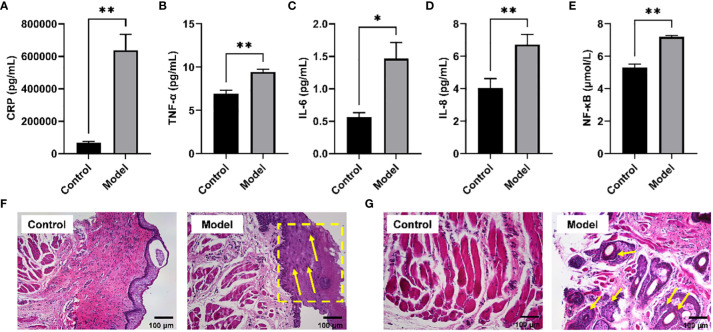
CIH induction increased typical pro-inflammatory mediators in serum and disrupted the morphological structure of the soft palate tissue. **(A)** CRP. **(B)** TNF-α. **(C)** IL-6. **(D)** IL-8. **(E)** NF-κB. **(F, G)** Histological analysis of mucosa and connective tissue. CIH, chronic intermittent hypoxia. The asterisk represents a significant difference between the two groups. *, p <0.05; **, p < 0.01.

It has been reported that long-term CIH can promote a sharp increase in the levels of pro-inflammatory cytokines, resulting in a systemic inflammatory state (21). Serum biochemical analysis showed that the levels of typical pro-inflammatory cytokines were significantly increased in the serum of rats exposed to CIH for 28 days, including h-CRP, TNF-α, IL -6, IL-8, and NF-κB (**Figures 1A–E**). Additionally, CIH induction contributed to the increased thickness of the soft palate mucosa with enlarged (yellow dotted box) and irregularly arranged squamous epithelial cells and loose connective tissue architecture (yellow arrow) (**Figure 1F**). Simultaneously, the significantly hyperplastic and hypertrophic soft palate glands infiltrated extensively into the muscles, resulting in the destruction and atrophy of the oropharyngeal muscles (yellow arrow) (**Figure 1G**). Our results showed that CIH induction could significantly induce the body’s inflammatory response, stimulate soft palate mucosal thickening and soft palate gland hyperplasia, and affect the expansion of the upper airway.

### 3.2 Effect of CIH induction on gut microbial diversity and bacterial composition

**Figure 2:**
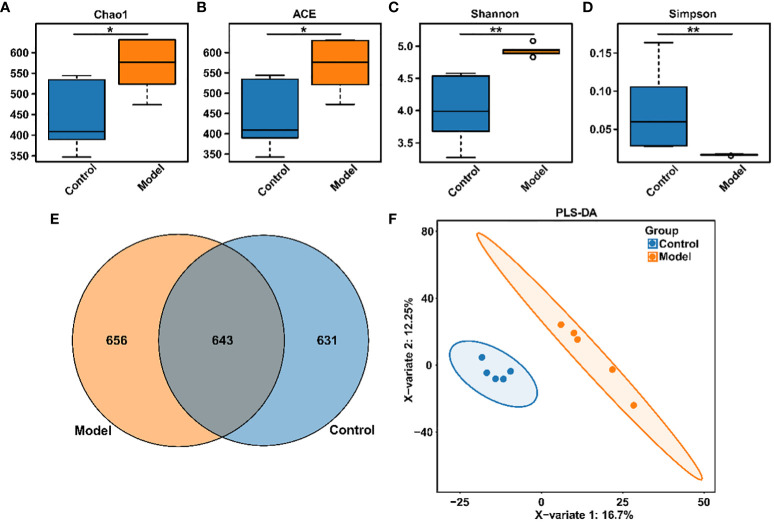
CIH induction altered gut microbiota diversity in rats. **(A)** Chao1 index. **(B)** ACE index. **(C)** Shannon index. **(D)** Simpson index. **(E)** Venn diagram. **(F)** PLS-DA diagram. The asterisk represents a significant difference between the two groups. *, p <0.05; **, p < 0.01.

Clinical studies have confirmed the presence of intestinal microbiota imbalance in OSA patients (22), and dysbacteriosis is considered to be a causative factor in the development of various complications associated with OSA (23). Given these reports, the gut microbial diversity and bacterial composition of CIH-induced rats were analyzed. The Accu16STM bacteria absolute quantitative high-throughput sequencing method was utilized to sequence the microbiota in fecal samples of rats, because this could reflect the real number of each microorganism in the sample and the real difference between the samples in the group (24). As shown in **Figures 2A–D**, Chao1, and ACE indexes, reflecting the community richness, were significantly increased in the CIH-induced group (Model group). Moreover, Shannon and Simpson indexes, reflecting the community diversity, were noticeably increased and decreased, respectively, in the Model group. This result revealed that CIH induced a significant difference in the α-diversity of gut microbiota. The Venn diagram (**Figure 2E**) showed 643 mutual OTUs in the Model and Control groups, and 656 and 631 peculiar OTUs in the two groups, respectively. The Partial least squares discrimination analysis (PLS-DA) analysis, which reflects β-diversity (**Figure 2F**), demonstrated a clear differentiation in the gut microbial community between the Model and Control groups, indicating a complete separation.

**Figure 3:**
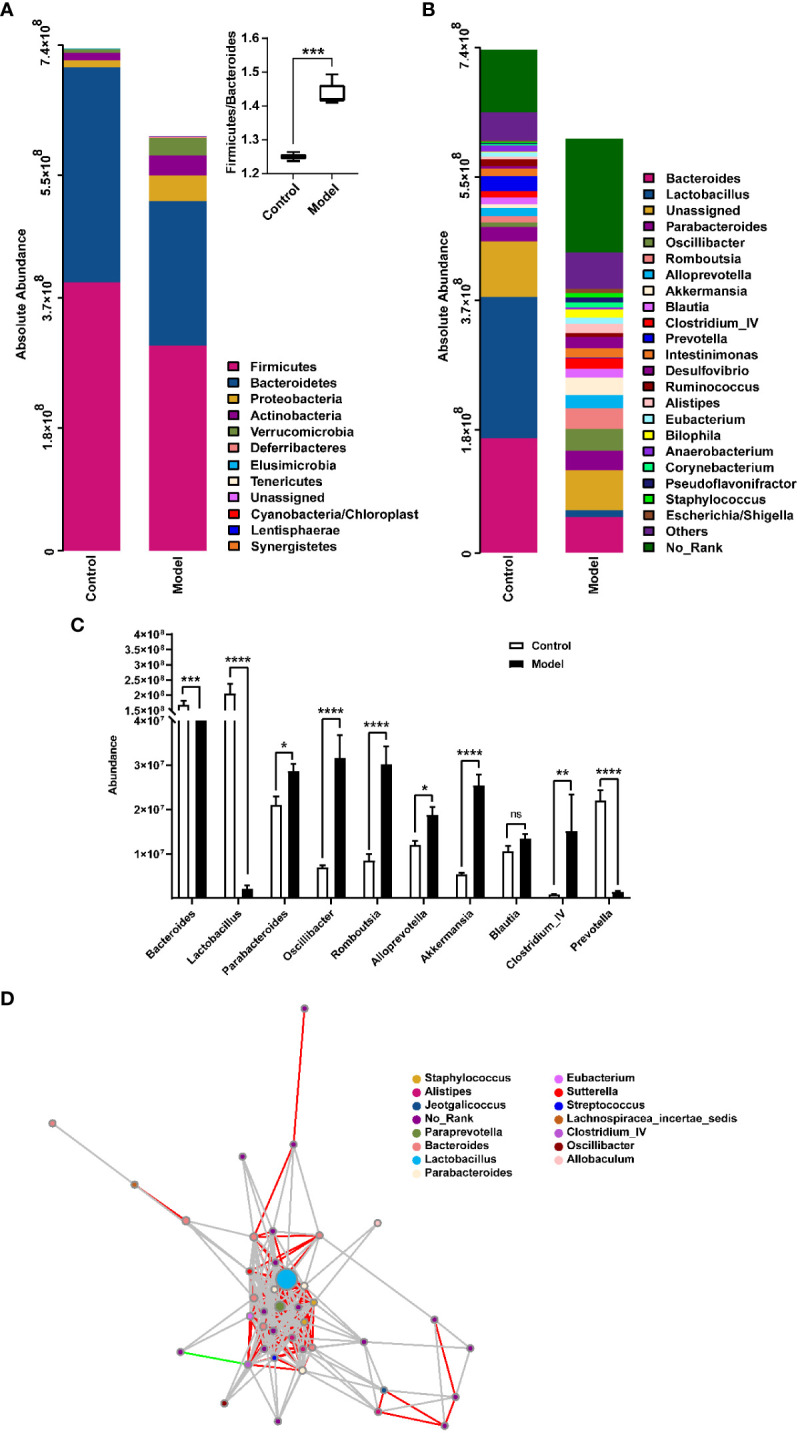
Histograms showing the relative distribution of gut microbiota in rat fecal samples were analyzed at the phylum **(A)** and genus **(B)** levels, indicating that CIH induction resulted in significant alterations in the composition and distribution of gut microbiota. **(C)** Quantitative abundance analysis of top 10 genera in rat fecal gut microbes. **(D)** A microbial interaction network diagram at the genus level was constructed based on Spearman correlation analysis. Each dot represents a species, and the size of the dot reflects its abundance. Different colors represent different species. Strong positive correlations (rho ≥ 0.6) are represented by red lines, strong negative correlations (rho ≤ -0.6) are represented by green lines, and weak correlations (|rho| < 0.6) are represented by gray lines. Color-coded annotations are based on the genus level. CIH, chronic intermittent hypoxia. The asterisk represents a significant difference between the two groups. *, p < 0.05; **, p < 0.01; ***, p < 0.001; ****, p < 0.0001. ns, not significant.

**Figure 4:**
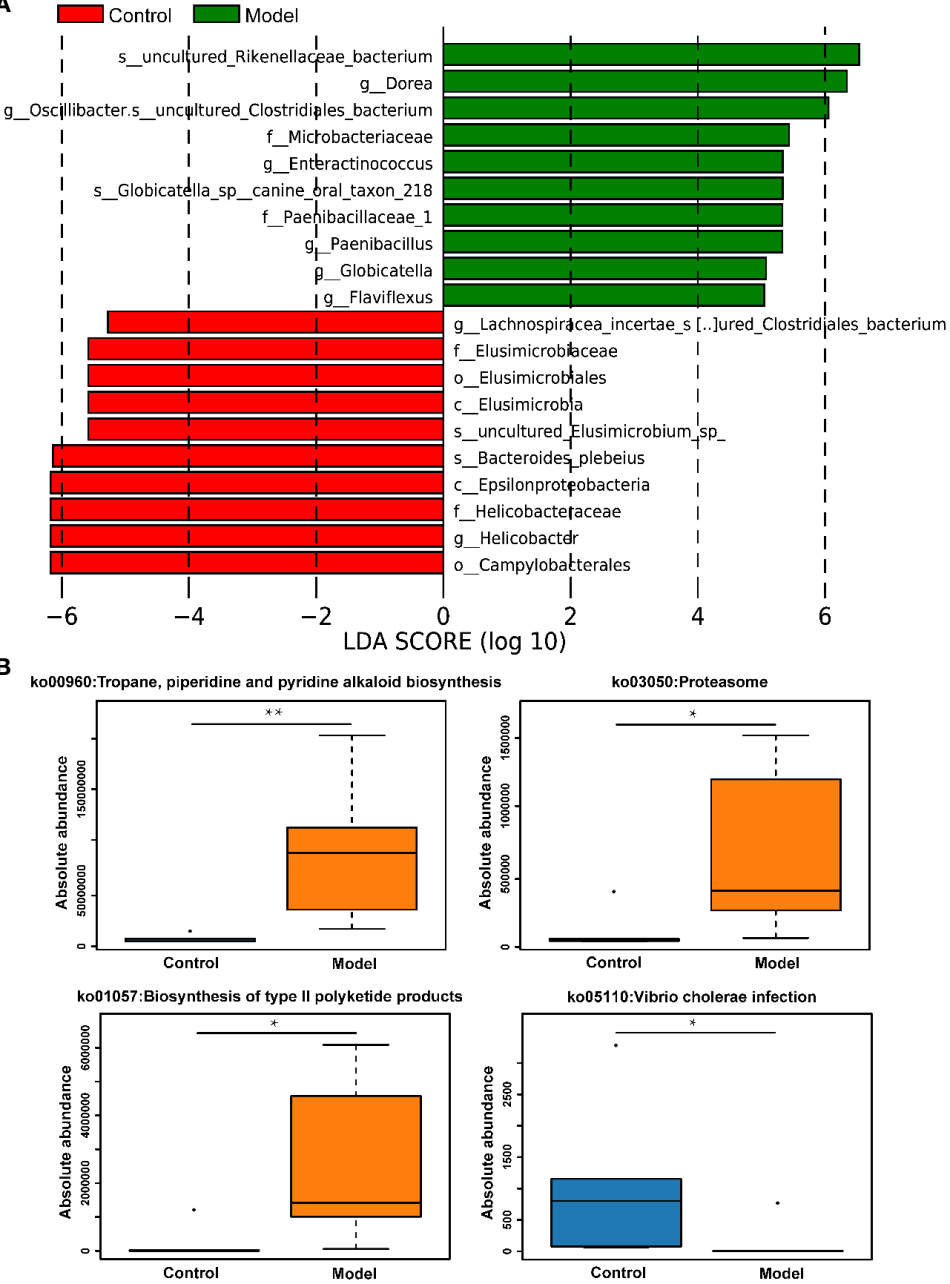
CIH induction specifically enriched certain gut microbes and regulated metabolic pathways of gut microbiota. **(A)** LDA discriminant histogram of the top 10 bacteria with the greatest diversity in enrichment. Bars of different colors represent different species with an LDA score (log10) greater than 4 in different groups and significantly high abundance in this group, and the length of the bar represents the value of the LDA score. **(B)** KEGG pathway enrichment analysis. The asterisk represents a significant difference between the two groups. *, p <0.05; **, p < 0.01.

The distribution of rat fecal microbiota was determined at both the phylum and genus levels to evaluate the potential impact of CIH induction on the gut microbiota. The results obtained from the preliminary composition analysis are presented in **Figure 3**. As could be seen from **Figure 3A**, *Firmicutes* and *Bacteroidetes* were the dominant bacteria at the phylum level, contributing to 49.47% and 34.92% of the total microbiota in the Model group, and 53.39% and 42.77% of total microbiota in the Control group, respectively. Additionally, a significant difference was observed in the *Firmicutes*/*Bacteroidetes* (F/B) ratio between the Model and Control groups. Further analysis showed that the absolute abundance of *Actinobacteria* and *Proteobacteria* of the Model group was greatly higher than that of the Control group. Interestingly, *Verrucomicrobia* was only observed in the Model group. At the genus level, the composition of rat fecal microbiota greater than 1% is shown in **Figure 3B**. The top ten fecal genera were *Bacteroides*, *Lactobacillus*, *Parabacteroides*, *Oscillibacter*, *Romboutsia*, *Alloprevotella*, *Akkermansia*, *Blautia*, *Clostridium_IV*, *Prevotella*, and their absolute abundance accounted for over 60%. However, significant differences were observed in the absolute abundance of microbial genera between the Model and Control groups, as depicted in **Figure 3C**. Specifically, CIH induction remarkably decreased the absolute abundance of *Bacteroides*, *Lactobacillus*, and *Prevotella*, and significantly increased the absolute abundance of *Parabacteroides*, *Oscillibacter*, *Romboutsia*, *Alloprevotella*, *Akkermansia*, and *Clostridium_IV*. Spearman correlation analysis based on the absolute abundance revealed the presence of antagonistic or synergistic effects among various microbial genera in rats from both the Model and Control groups, as illustrated in **Figure 3D**. In addition to top genera, linear discriminant analysis (LDA) revealed that the intestinal microbiota of rats in the model group specifically and significantly enriched *Dorea*, *Oscillibacter*, *Enteractinococcus*, *Paenibacillus*, *Globicatella*, and *Flaviflexus* genera (**Figure 4A**).

In addition to assessing changes in the absolute abundance of microorganisms in rat feces, the effects of CIH induction on KEGG pathways involved in the metabolism of microorganisms were also assessed. It was apparent from **Figure 4B** that CIH induction significantly activated metabolic pathways involving microbes including the tropane, piperidine and pyridine alkaloid biosynthesis, proteasome, and biosynthesis of type II polyketide products. Additionally, CIH induction significantly inhibited the pathway of vibrio cholerae infection.

### 3.3 Effect of CIH induction on metabolic profiles in serum and soft palate tissue

**Figure 5:**
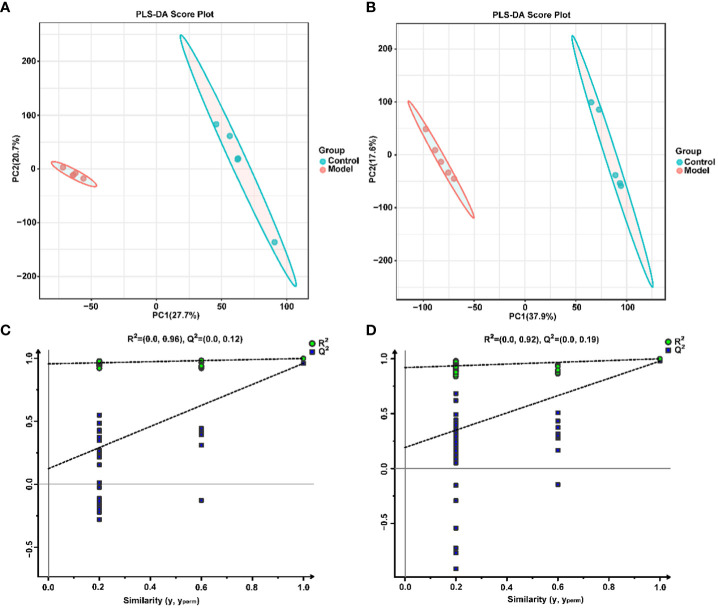
Multivariate statistical analysis of serum metabolites. PLS-DA score plot in positive **(A)** and negative **(B)** ion modes. Permutation tests of the PLS-DA model with 200 cycles in positive **(C)** and negative **(D)** ion modes.

**Figure 6:**
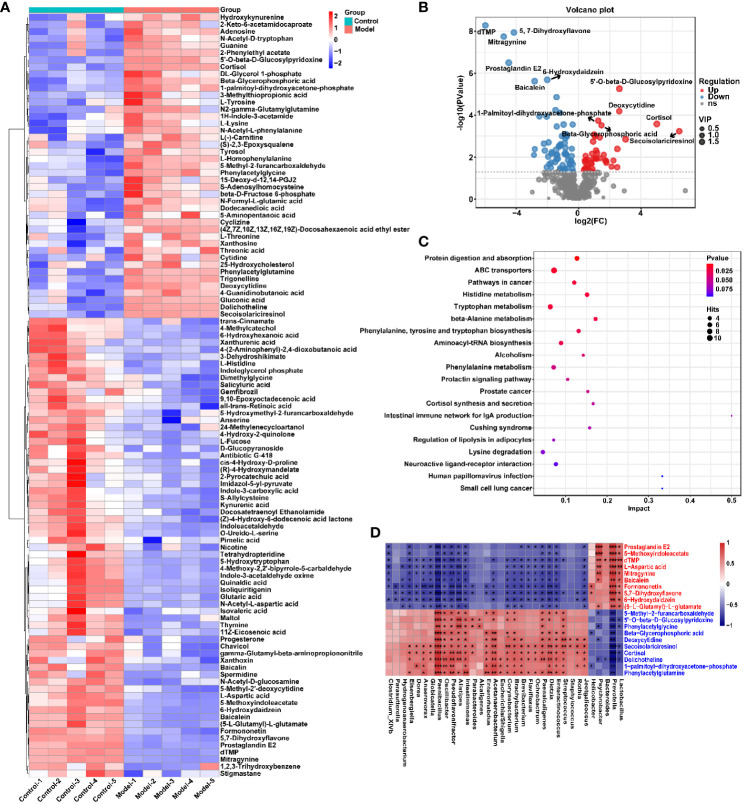
CIH induction significantly affected the serum metabolic profile of rats. **(A)** Cluster analysis heat map of CIH-induced differential metabolites. **(B)** Volcano plot of differential metabolites in serum. In the graph, each data point represents a metabolite. The x-axis represents the logarithmic value of Log2 of the quantitative difference of a specific metabolite, while the y-axis represents the logarithmic value of -log10 of the p-value. By default, the top 6 metabolites with the smallest p-values are displayed. **(C)** Bubble plot of influencing factors of metabolic pathways. In the graph, each data point represents a metabolic pathway. The x-axis represents the impact value enriched in different metabolic pathways, while the y-axis represents the enriched pathway. Dots on the graph indicate the number of corresponding metabolic molecules associated with each pathway. Colors are related to p-values. **(D)** Correlation heatmap between the top 10 up-regulated/down-regulated metabolites and the gut microbiota. The asterisk represents a significant difference between the two groups. *, p < 0.05; **, p < 0.01; ***, p < 0.001.

To explore the effect of CIH induction on rat serum metabolic profiles, UHPLC-MS-based global analysis of untargeted metabolomics was performed in positive and negative ionization modes. The total ion chromatograms of the samples were well overlaid (**Supplementary Figure S1**), indicating favorable reproducibility of the LC-MS analysis. PLS-DA can maximize group differences and facilitate the search for differential metabolites. The clustering characteristics between the Model and Control groups were characterized by PLS-DA, and there was a clear separation between the two groups in both positive and negative ionization modes (**Figures 5A, B**), which indicated that CIH induction perturbed the metabolism of rats. The permutation test was employed to verify the effectiveness of the PLS-DA model. **Figures 5C, D** showed that the values of all Q^2^ points (blue) and R^2^ points (green) from left to right were lower than the value of the original blue Q^2^ point and green R^2^ point on the far right in both positive ion and negative ion modes, proving a reliable and not overfitting model. In this study, potential perturbed biomarkers in rat serum after CIH induction were screened based on variable importance for the projection (VIP) values (VIP>1) and significance discriminant p-values (p<0.05). **Figure 6A**; **Supplementary Table S1** showed that there were significant differences in 108 metabolites in rat serum after CIH perturbation, which were considered potential biomarkers and could be mainly classified as amino acids, organic acids, and a few flavonoids or sterols. A significant decrease in 65 metabolites was observed in the Model group compared to the Control group while a significant increase in 43 metabolites was observed. Among the significantly decreased metabolites, the top 10 substances with the most significant difference (**Figure 6B**) were thymidylate (dTMP), 5,7-dihydroxyflavone, mitragynine, prostaglandin E2, 6-hydroxydaidzein, baicalein, formononetin, (5-L-Glutamyl) -L-glutamate, L-aspartic acid, and 5-methoxyindoleacetate. Among the significantly elevated metabolites, the top 10 most significantly different metabolites (**Figure 6B**) were 5’-O-beta-D-glucosylpyridoxine, deoxycytidine, 1-palmitoyl-dihydroxyacetone-phosphate, cortisol, beta-glycerophosphoric acid, secoisolariciresinol, 5-Methyl-2-furancarboxaldehyde, phenylacetylglycine, phenylacetylglutamine, and dolichotheline. According to the significantly changed serum metabolites after CIH induction, 12 metabolic pathways with an impact value greater than 0.15 were observed to be significantly affected, including protein digestion and absorption, ABC transporters, pathways in cancer, histidine metabolism+B5:O5, tryptophan metabolism, beta-alanine metabolism, phenylalanine, tyrosine and tryptophan biosynthesis, aminoacyl-tRNA biosynthesis, alcoholism, phenylalanine metabolism, prolactin signaling pathway, and prostate cancer (**Figure 6C**; **Supplementary Table S2**). In addition, these metabolites were found to be significantly associated with the abundance changes of *Helicobacter*, *Psychrobacter*, *Bacteroides*, *Prevotella*, *Lactobacillus*, and other genera (**Figure 6D**). Taken together, CIH induction might affect these metabolites and their metabolic pathways in serum by regulating the gut microbiota structure.

**Figure 7:**
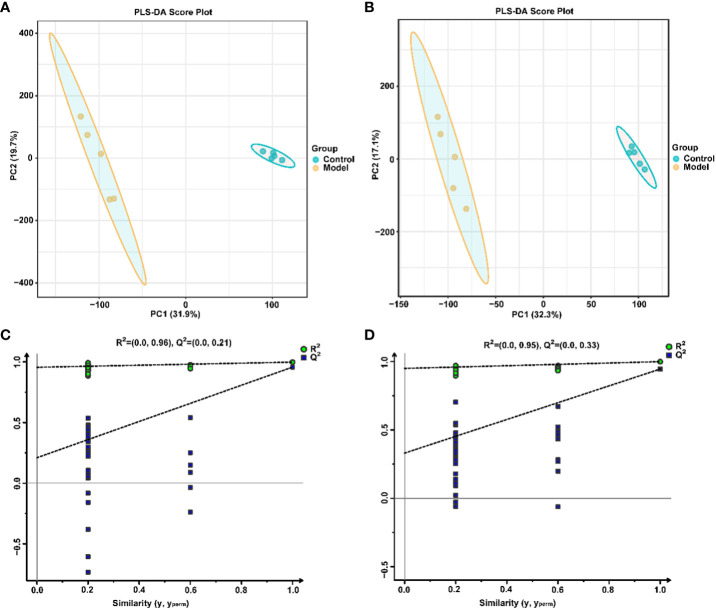
Multivariate statistical analysis of soft palate tissue metabolites. PLS-DAscore plot in positive **(A)** and negative **(B)** ion modes. Permutation tests of the PLS-DA model with 200 cycles in positive **(C)** and negative **(D)** ion modes.

**Figure 8:**
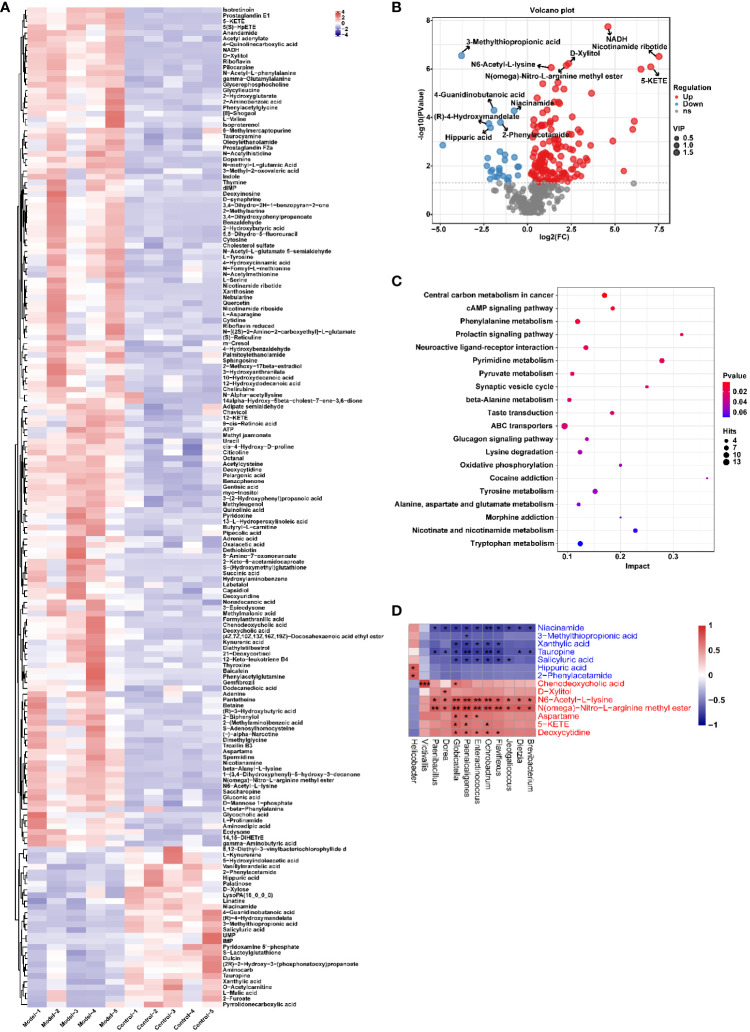
CIH induction significantly affected the metabolic profile of the rat soft palate tissues. **(A)** Cluster analysis heat map of CIH-induced differential metabolites. **(B)** Volcano plot of differential metabolites in serum. In the plot, each data point represents a metabolite, with the x-axis showing the logarithmic value of the log2 fold change of a specific metabolite, and the y-axis showing the logarithmic value of the negative logarithm (base 10) of the p-value. Up-regulated differentially expressed metabolites are represented by red dots, down-regulated metabolites are represented by blue dots, and metabolites that are detected but do not meet the filtering parameters are represented by gray dots. By default, the plot displays the top 6 metabolites with the smallest p-values. **(C)** Bubble plot of influencing factors of metabolic pathways. In the plot, each data point represents a metabolic pathway, with the x-axis representing the impact value enriched in different metabolic pathways and the y-axis representing the enriched pathway. The dots on the plot indicate the number of corresponding metabolic molecules present in each pathway. Colors are related to p-values. **(D)** Correlation heatmap between the top 10 up-regulated/down-regulated metabolites and the gut microbiota. The asterisk represents a significant difference between the two groups. *, p <0.05; **, p < 0.01; ***, p < 0.001.

**Figure 9:**
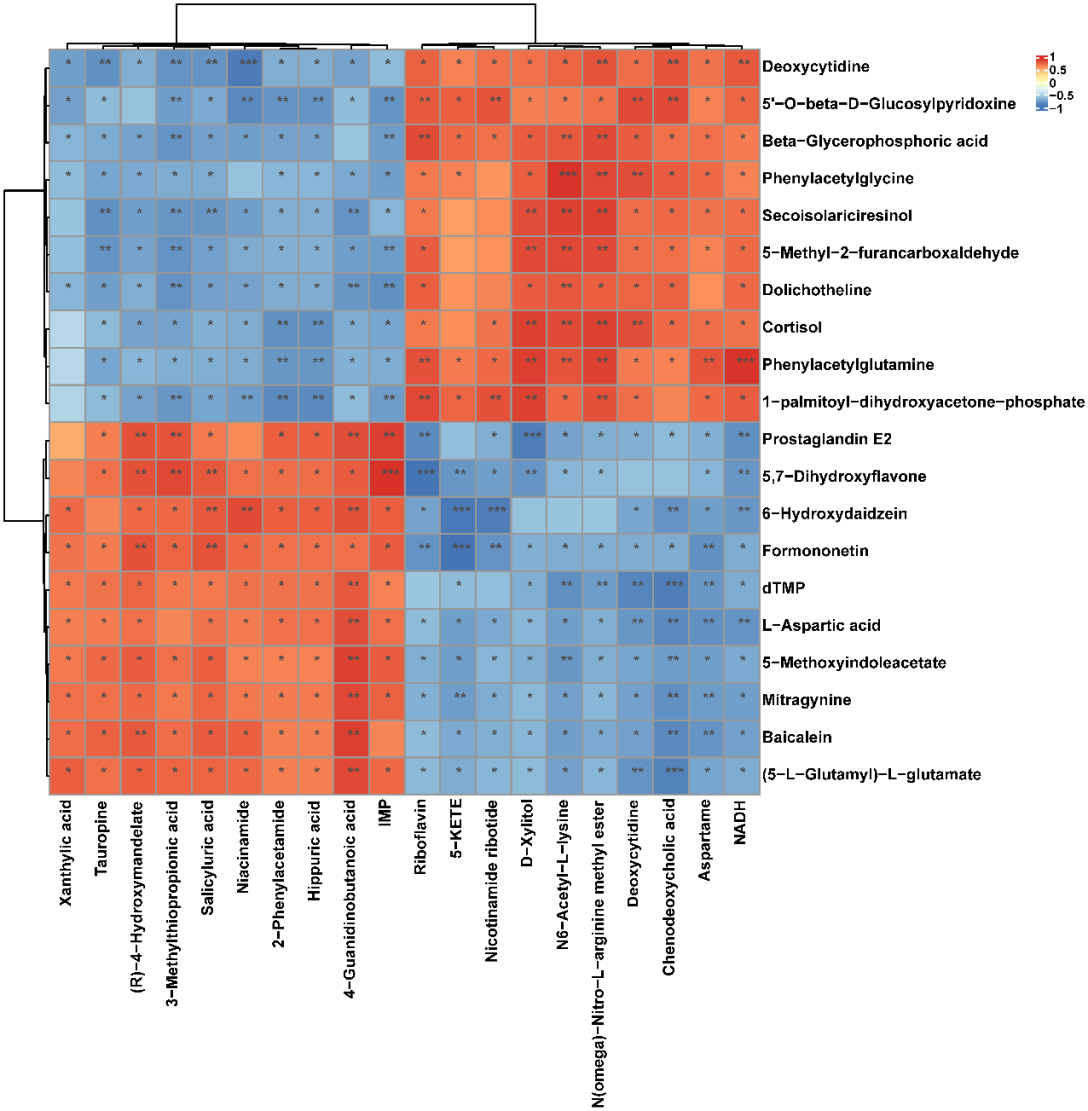
Correlation heatmap between the top 10 up-regulated/down-regulated metabolites in serum and the top 10 up-regulated/down-regulated metabolites in soft palate tissue. The asterisk represents a significant difference between the two groups. *, p <0.05; **, p < 0.01; ***, p < 0.001.

Similarly, the effect of CIH induction on the metabolic profile of soft palate tissue was analyzed. The total ion chromatograms of samples based on positive and negative ionization modes were well overlapped (**Supplementary Figure S2**), whereas PLS-DA characterization analysis showed that both groups were separated in positive/negative ionization modes (**Figures 7A, B**). In addition, the permutation test of the PLS-DA model (**Figures 7C, D**) showed the reliability of the model. Next, 175 potential differential metabolites, such as NADH, nicotinamide ribotide, and D-xylitol, were screened out in rat soft palate tissues after CIH induction according to VIP>1 and p<0.05 (**Figure 8A**; **Supplementary Table S3**). Compared with the Control group, 147 metabolites were significantly decreased and 28 metabolites were significantly increased in the model group. Among the significantly reduced metabolites, the top 10 metabolites with the most significant differences (**Figure 8B**) were 3-methylthiopropionic acid, 4-guanidinobutanoic acid, niacinamide, (R)-4-hydroxymandelate, 2-phenylacetamide, hippuric acid, IMP, salicyluric acid, tauropine, and xanthylic acid. Among the significantly elevated metabolites, the top 10 metabolites with the most significant differences (**Figure 8B**) were NADH, nicotinamide ribotide, D-xylitol, N(omega)-nitro-L-arginine methyl ester, 5-KETE, N6 -acetyl-L-lysine, chenodeoxycholic acid, riboflavin, deoxycytidine, and aspartame. In addition, these metabolites were found to be significantly correlated with the top 10 signature metabolites in serum (**Figure 9**). According to the significant changes in rat soft palate metabolites after CIH induction, 15 metabolic pathways with an impact value greater than 0.10 were observed to be significantly affected, including central carbon metabolism in cancer, cAMP signaling pathway, phenylalanine metabolism, prolactin signaling pathway, neuroactive ligand-receptor interaction, pyrimidine metabolism, pyruvate metabolism, synaptic vesicle cycle, beta-alanine metabolism, taste transduction, ABC transporters, glucagon signaling pathway, lysine degradation, oxidative phosphorylation, and cocaine addiction (**Figure 8C**; **Supplementary Table S4**). Furthermore, these metabolites were found to be significantly associated with the abundance changes of *Elusimerbium*, *Helicobacter*, *Victivallis*, *Paenibacillus*, *Dorea* genera, etc. (**Figure 8D**). Taken together, CIH induction might affect these metabolites and their metabolic pathways in soft palate tissues, and these changes might be related to alterations in the structure of the gut microbiota.

### 3.4 Effect of CIH induction on the cAMP-related pathway in soft palate tissue

**Figure 10:**
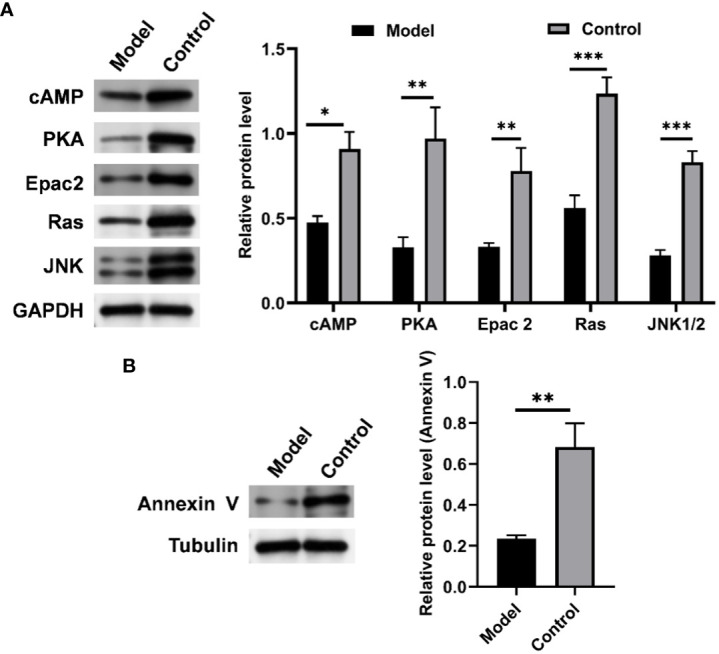
CIH induction significantly down-regulated the expression of cAMP-related pathways in soft palate tissue. **(A)** Relative protein expression of cAMP, PKA, Epac2, Ras, and JNK. **(B)** Relative protein expression of Annexin V. The asterisk represents a significant difference between the two groups. *, p <0.05; **, p < 0.01; ***, p < 0.001.

To further verify the role of the cAMP pathway in CIH-simulated OSA (**Supplementary Figure S3**), the expression levels of key proteins in the cAMP pathway in soft palate tissue were analyzed. As shown in **Figure 10**, CIH induction triggered a significant decrease in the protein expression of cAMP, PKA, Epac2, Ras, and JNK1/2. Meanwhile, the protein expression of annexin V was also markedly up-regulated by CIH induction. This result was consistent with the KEGG pathway enrichment analysis in the metabolomics assay of soft palate tissue, which further validated the protruding role of the cAMP pathway in CIH-simulated OSA.

## 4 Discussion

OSA is characterized by recurring episodes during sleep where the upper airway narrows or collapses, leading to decreased ventilation or apnea. Apnea and hypopnea lead to desaturation of blood oxygen, increased carbon dioxide in the blood, and subsequent interruption of wakefulness and sleep due to repeated activation of the central nervous system. Therefore, CIH is considered a key mechanism of endothelial dysfunction and increased cardiovascular disease risk in patients with OSA. Patients with OSA are at relatively high risk of developing cardiovascular diseases such as ischemic heart disease, heart failure, cardiac arrhythmias, stroke, and transient ischemic attack (25). OSA has become a serious public health issue due to its high prevalence, lack of access to diagnostic procedures, and association with social, occupational, and cardiovascular risk complications (26). Exploring the molecular mechanism of the occurrence and development of OSA and identifying early diagnostic indicators are extremely important for the clinical intervention and treatment of OSA.

In this study, a rat model simulating the process of OSA simulated by CIH was successfully established to explore the underlying mechanism of OSA pathogenesis under chronic hypoxic conditions. CIH stimulates the overproduction of reactive oxygen species (ROS), which is key to its pro-inflammatory response (21). Secondary to ROS expression, the levels of some pro-inflammatory cytokines increase, producing a systemic inflammatory state. Nocturnal hypoxia has been reported to lead to higher concentrations of h-CRP (27), while several other studies have shown that the levels of h-CRP, IL-6, and IL-8 in OSA patients were significantly increased (28, 29). Indeed, systemic inflammation is one of the main underlying pathological mechanisms linking OSA to cardiovascular disease. As expected, we found that CIH induction significantly elevated the levels of several canonical pro-inflammatory cytokines. Consistently, we also found thickening of the soft palate mucosal layer, muscle atrophy consistent with glandular hyperplasia, and soft palate connective tissue loosening in a CIH-induced rat model. Clinical studies have revealed that the inflammatory cascade had a marked effect on the vascular region, which could activate the sympathetic nervous system and produce endothelial dysfunction (30, 31). Although muscular changes are considered an adaptive compensation for snoring in OSA patients (32), atrophy of this musculature remains ineffective against pharyngeal collapse. The velopharyngeal muscle plays a crucial role in regulating the upper airway, and understanding its pathological changes can help ameliorate the development of OSA and its complications. Possible explanations for the changes in muscles exposed to CIH are systemic inflammation-induced neuronal damage of the soft palate or endothelial cell dysfunction.

CIH-mediated intestinal dysbiosis has been implicated in the pathophysiology of OSA and related diseases. Evidence of intestinal microbial dysbiosis has been observed in animal models of OSA (33, 34). However, most related studies have focused solely on the relative abundance of gut microbiota, which has limited capacity to reveal the complex interactions between microbes and host health. To fully investigate host-microbe interactions, it is essential to turn relative quantification into absolute quantification (35). In our current study, we utilized the Accu16STM bacterial absolute quantitative high-throughput sequencing method to comprehensively reflect the potential impact of bacterial community changes on the host. Our results showed that CIH induction significantly increased the overall number and diversity of the gut microbiota, consistent with previous reports (36). At the phylum level, CIH induction increased the F/B ratio, a marker of gut dysbiosis, which had been associated with disease states (23). Moreover, CIH induction specifically promoted the colonization of *Verrucomicrobia* in the gut. Currently, few functional studies have been conducted on *Verrucomicrobia*, further research is needed to elucidate its specific role in the gut. Significant differences in the abundance of microbiota were observed at the phylum and genus levels in response to CIH exposure. It is worth noting that in an ecosystem, not all species hold the same importance, and the dominance of microorganisms within the gut micro-ecosystem can greatly affect the gut microenvironment (12). Among the top 10 microbial genera that accounted for over 1% of abundance, the abundance of *Bacteroides*, *Lactobacillus*, and *Prevotella* was significantly reduced, while the abundance of *Parabacteroides*, *Oscillibacter*, *Romboutsia*, *Alloprevotella*, *Akkermansia*, and *Clostridium_IV* was significantly increased. These findings indicate a relative increase in obligate anaerobes and a substantial decrease in beneficial bacteria following intermittent hypoxic exposure. The reduction in intestinal oxygen levels under intermittent hypoxia confers an ecological selective advantage on obligate anaerobes, making them more competitive and able to overgrow. In contrast, facultative anaerobes and even aerobic bacteria are at a disadvantage. The *Bacteroides* genus, which primarily inhabits the colon, plays a crucial role in carbohydrate and fiber fermentation, producing short-chain fatty acids (SCFAs) such as butyrate, acetate, and propionate (37). SCFAs are essential in maintaining human health by providing the primary source of nutrition and energy for colon cells, protecting the intestinal mucosal barrier, reducing inflammation in the host, and enhancing intestinal peristalsis (38). Interestingly, SCFAs also have direct anti-inflammatory effects on the gut, contribute to mucin synthesis, reduce bacterial translocation, maintain gut integrity, and reduce gut inflammation (39). *Lactobacillus* genus, a beneficial gut bacterium, can improve gut health and show anti-inflammatory, anti-diabetic, and anti-obesity effects (40). New evidence suggests that *Lactobacillus* may also help improve sleep and reduce the effects of sleep deprivation (41). *Prevotella* can effectively decompose dietary fiber and plant polysaccharides in the gut, producing SCFAs with potential benefits in maintaining host glucose homeostasis and regulating host metabolism (41). *Parabacteroides* have been reported to be closely related to body inflammation, endotoxin production, increased risk of death, and antibiotic resistance (42). *Oscillibacter*, *Romboutsia*, and *Clostridium IV* were found in obese-prone insulin-resistant mice and were absent in their obese-resistant hosts (43, 44). *Alloprevotella* was significantly enriched in the gut of colitis mice (45), and *Akkermansia* was recognized as a conditional therapeutic bacteria associated with hyperglycemia (46). Rodent studies have shown that sleep fragmentation leads to increased food intake, visceral adiposity, inflammation, and insulin resistance, while chronic sleep deprivation results in altered energy metabolism (47, 48). In response to these changes, the gut microbiota involved in these metabolic regulations responds positively. Specifically, *Dorea*, *Enteractinococcus*, *Paenibacillus*, *Globicatella*, and *Flaviflexus* genera were found to be enriched in the gut of rats chronically exposed to intermittent hypoxia, and these genera were previously reported to be associated with sleep fragmentation related to sleep disorders (41, 49, 50). Furthermore, CIH induction significantly enhanced the functions of tropane, piperidine, and pyridine alkaloid biosynthesis, proteasome, and biosynthesis of type II polyketide products of gut microbiota. Tropane, piperidine, and pyridine alkaloid biosynthesis were reported to be related to cytotoxicity (51). As for tropane, piperidine and pyridine alkaloid biosynthesis, more reports are needed to further clarify their effects on host health. Collectively, CIH induction resulted in a decrease in beneficial bacteria producing SCFAs in the gut, an increase in harmful bacteria associated with inflammation, and an enrichment of microbiota associated with sleep disturbance/sleep fragmentation.

Recently, there has been an interest in investigating changes in the serum metabolome of patients with OSA. Changes in the gut microbiota composition due to CIH may result in altered serum metabolomes. Our results showed that a majority of the serum differential metabolites that are affected by CIH are amino acids, organic acids, and a few flavonoids or sterols. Amino acids and organic acids are important components of the serum metabolome and are responsible for various essential functions in the body, such as energy regulation, redox balance, biosynthesis, and normal metabolism (52, 53). Since it was difficult to list all 108 differential metabolites in serum, the top 10 significantly decreased and the top 10 significantly increased metabolites compared with Control were listed and analyzed. The levels of these characteristic metabolites were found to be significantly altered in response to changes in the composition and structure of the gut microbiota, and their effects on bioprocesses primarily focused on inflammation, oxidative stress, depression, cancer, and cardiovascular disease, which were all involved in the development of OSA pathophysiology (21, 54). For example, thymidylate (dTMP) and deoxycytidine are involved in DNA synthesis, and an imbalance in their levels may lead to excessive production of ROS, further inducing cell apoptosis (55). Deoxyribonucleosides have been used as biomarkers of oxidative stress, and their levels were found to be higher in patients with severe OSA (54). Some of the significantly altered metabolites, such as 5,7-dihydroxyflavone, mitragynine, prostaglandin E2, 6-hydroxydaidzein, baicalein, formononetin, (5-L-Glutamyl)-L-glutamate, and L-aspartic acid, have been reported to exhibit favorable anti-inflammatory, antioxidant, and antidepressant activity, which can prevent the release of pro-inflammatory mediators and oxidative stress products, playing a crucial role in the regulation of nutrient metabolism, oxidative defense, and immune function (56–65). Other metabolites, such as 5-methoxyindoleacetate, cortisol, beta-glycerophosphoric acid, phenylacetylglycine, phenylacetylglutamine, and dolichotheline, have been associated with ischemic stroke, stress, osteogenic differentiation, cardiovascular disease, and amino acid synthesis or metabolism (66–70). However, the specific functions of some of the remaining metabolites could not be presented due to limited reports, and more studies were needed to reveal their potential regulatory effects. Through KEGG enrichment analysis, the top 10 pathways enriched by these differential metabolites were mainly related to protein transport, digestion and absorption, amino acid synthesis and metabolism, and cancer development. SCFAs are essential for colon function and body health, and they are produced during protein digestion and absorption (71). Amino acids play a key role in various fundamental functions in the human body and are closely related to OSA, and the gut microbiota plays a crucial role in intestinal protein/amino acid metabolism (72). Cancer development involves abnormal cell proliferation, which drives the uncontrolled metabolic demands of cells (73). Therefore, these findings suggest that serum differential metabolites involved in processes such as inflammation, oxidative stress, depression, cancer, and cardiovascular disease may act as clinical class-differentiated biomarkers of OSA.

Further analysis revealed that the changes in serum metabolites had an impact on the metabolism in soft palate tissue, which played a significant role in pathological development. Among the top 10 metabolites that were significantly reduced in the Model group, 4-guanidinobutanoic acid is a key metabolite found in the urine of rats exposed to carcinogenic heterocyclic arylamines, and its levels were significantly decreased in urine after exposure to such carcinogens (74). Niacinamide is known to contribute to cellular energy metabolism and defense system, and it exhibits excellent anti-oxidation, anti-cancer, and anti-aging effects (75). Hippuric acid is a metabolite that can distinguish between physical fitness and frailty, and low levels of plasma hippuric acid might be a potential marker of a frail state (76). Inosine monophosphate (IMP) serves as an intracellular precursor of adenosine monophosphate and guanosine monophosphate and plays a central role in intracellular purine metabolism. Studies have shown that IMP inhibits the production of tumor necrosis factor (TNF)-α and increases the production of IL-10 in endotoxemic mice (77). Salicylic acid has been found to possess strong antibacterial and antibiofilm properties. Tauropine is a mitochondrial matrix buffer that plays an indirect antioxidant role by stabilizing mitochondrial oxidation (78). Xanthylic acid is an intermediate product generated by the catabolism of guanine and adenosine triphosphate in muscle tissue, and the intracellular content of xanthine is correlated with cell activity (79). Moreover, its concentration levels in urine and plasma were indicators of certain pathological states such as cerebral ischemia (80). Additionally, 3-methylthiopropionic acid, (R)-4-hydroxymandelate, and 2-phenylacetamide were first reported to be significantly reduced in soft palate tissue following CIH induction, indicating their potential as targets for OSA intervention. Among the top 10 significantly increased metabolites, NADH, an important redox factor responsible for transferring electrons from the TCA cycle to the ETC to generate ATP, accumulates under hypoxic conditions where respiration is impaired and can damage cells leading to their death (81). Nicotinamide ribotide, a precursor of NAD^+^, reduces exercise performance in rats by altering redox homeostasis and causing cells to enter a suboptimal state (82). N(omega)-nitro-L-arginine methyl ester is a NO synthase inhibitor that significantly increases blood pressure, promoting the development of inflammation and fibrosis in organs/tissues (83). 5-keto eicosatetraenoic acid (5-KETE) is a chemotactic factor that attracts inflammatory cells to aggravate inflammation (84). N6-acetyl-L-lysine is a signature metabolite in the plasma of obese COVID-19 patients that regulates the metabolic enzyme activity of glucose and fatty acids (85). Chenodeoxycholic acid is identified as a novel biomarker of coronary artery calcification in nondiabetic hemodialysis patients, but its involvement in brown fat metabolism suggests a need for more research to clarify its function (86, 87). Deoxycytidine is a product of DNA oxidation, and its levels reflect the degree of DNA degradation. D-xylitol, aspartame, and riboflavin are exogenous metabolites that may originate from components or additives in rat diets.

KEGG analysis identified fifteen differentially enriched pathways, mainly involved in cancer development, signaling pathways, amino acid metabolism, nucleotide precursor or intermediate metabolism, respiratory process, and disease. CIH-induced changes in signaling pathways and amino acid metabolism disorders involved abnormal respiratory processes and abnormal proliferation of soft palate glands and thickening of the mucosal layer, accompanied by the formation of inflammation (21, 54, 72, 73). Participation of specific products also aggravated the pathological development of soft palate tissue, such as significantly higher urinary 8-Hydroxy-2-deoxyguanosine excretion observed in patients with severe OSA (88). Interestingly, a significant enrichment of the cAMP pathway was observed, providing interesting ideas for the treatment of OSA. Overall, our findings suggest that CIH induction significantly affects the production of metabolites involved in cancer development, signaling pathways, and amino acid metabolism, and these differential metabolites might serve as potential targets for intervention or treatment of OSA.

cAMP is the first identified second messenger, which has a fundamental role in the cellular response to many extracellular stimuli. Multiple studies have demonstrated that the cAMP-mediated signaling pathway plays a significant role in the development and progression of various complications induced by CIH (89, 90). For example, Li et al. found that salidroside could alleviate the overproduction of ROS and endothelial barrier damage in CIH-induced atherosclerosis mice by activating the cAMP pathway (90). In addition, Pan et al. indicated that inhibition of phosphodiesterase 4B could attenuate CIH-induced pulmonary hypertension by regulating cAMP signaling (89). Similar to these reports, our results showed that CIH-induced dysregulation of the gut microbiota and alteration of the metabolic profile might be associated with the downregulation of the cAMP pathway expression in rat soft palate tissue. Accumulating literature has shown that activation of the cAMP pathway positively regulates mitochondrial biogenesis (91), which is further involved in the regulation of endothelial/myocyte homeostasis (92). Our results showed that the downregulation of the cAMP pathway was accompanied by inhibition of annexin V expression. Previous studies have shown that annexin V as a sensory nerve marker contributed to the collapse of the upper airway soft palate innervated by supporting muscles (20). Therefore, our result suggested that CIH induction might partly suppress the expression of annexin V by inhibiting the expression of the cAMP pathway, thereby promoting the collapse of the upper airway soft palate. However, the cascade pathways closely related to cAMP and their interaction mechanism with annexin V should also be investigated in the future.

## 5 Conclusion

In summary, this study demonstrates that CIH induction leads to inflammation and histopathological injury in the body, particularly in the soft palate. Moreover, CIH induction influenced the composition and structure of the gut microbiota, and it also significantly altered the metabolic profiles in both serum and soft palate tissue. Furthermore, targeting the cAMP pathway may hold promise for interventions or treatments for OSA. However, the current study did not directly establish a connection between the gut microbiota and the serum and tissue metabolomes. Subsequent experiments involving fecal transplantation in germ-free or gnotobiotic mice are required to confirm this link.

## Data availability statement

The original contributions presented in the study are publicly available. This data can be found here: 10.6084/m9.figshare.23587119 and 10.6084/m9.figshare.23587164 (figshare).

## Ethics statement

The animal study was approved by Institutional Animal Use and Care Committee guidelines of the Second Military Medical University. The study was conducted in accordance with the local legislation and institutional requirements.

## Author contributions

CL: Conceptualization, methodology, software, writing—original draft preparation, and writing—review and editing. SS: supervision, project administration, and funding acquisition. All authors have read and agreed to the published version of the manuscript.
