# Supplementary material for: Gut microbiota and metabolic profiles in chronic intermittent hypoxia-induced rats: disease-associated dysbiosis and metabolic disturbances
Source: Front Endocrinol (Lausanne). 2024 Jan 12;14:1224396. doi: 10.3389/fendo.2023.1224396 (PMC10811599; doi:10.3389/fendo.2023.1224396)
Supplement: Supplementary file 1 [file DataSheet_1.docx]

**Supplementary materials**


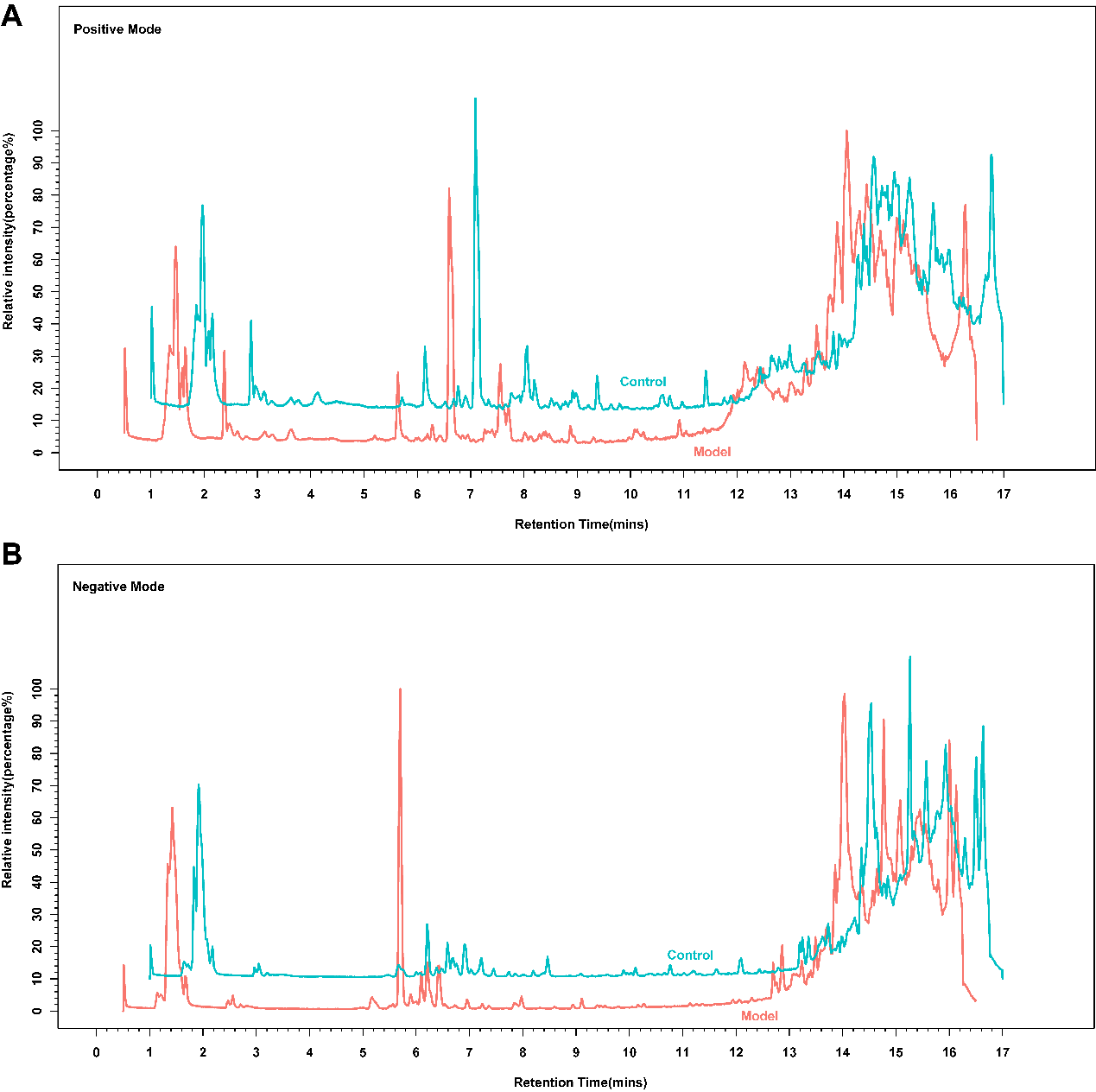


**Figure S1.** Total ion chromatogram of rat serum samples in positive (A) and negative (B) models.


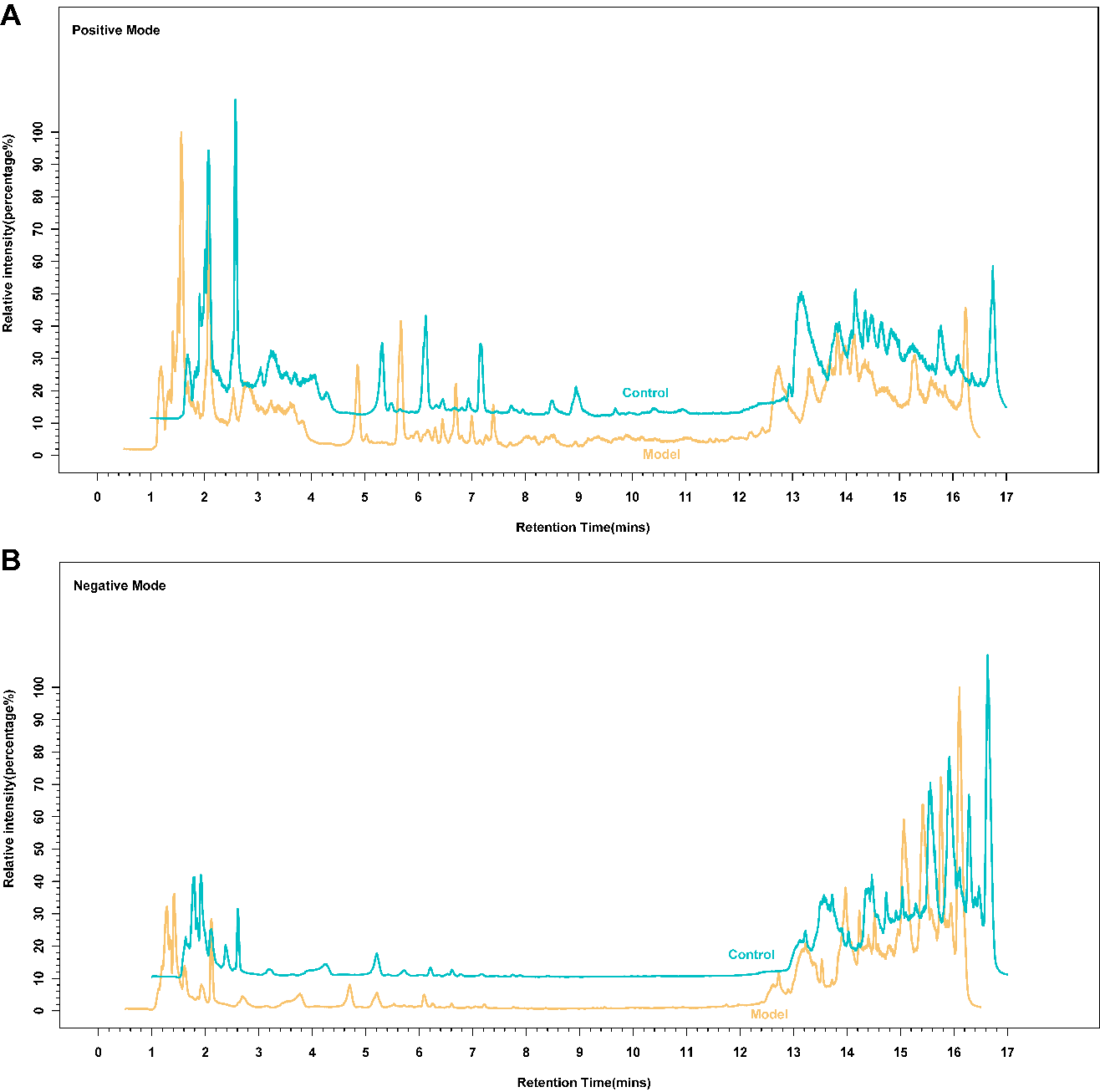


**Figure S2.** Total ion chromatogram of rat soft palate samples in positive (A) and negative (B) models.


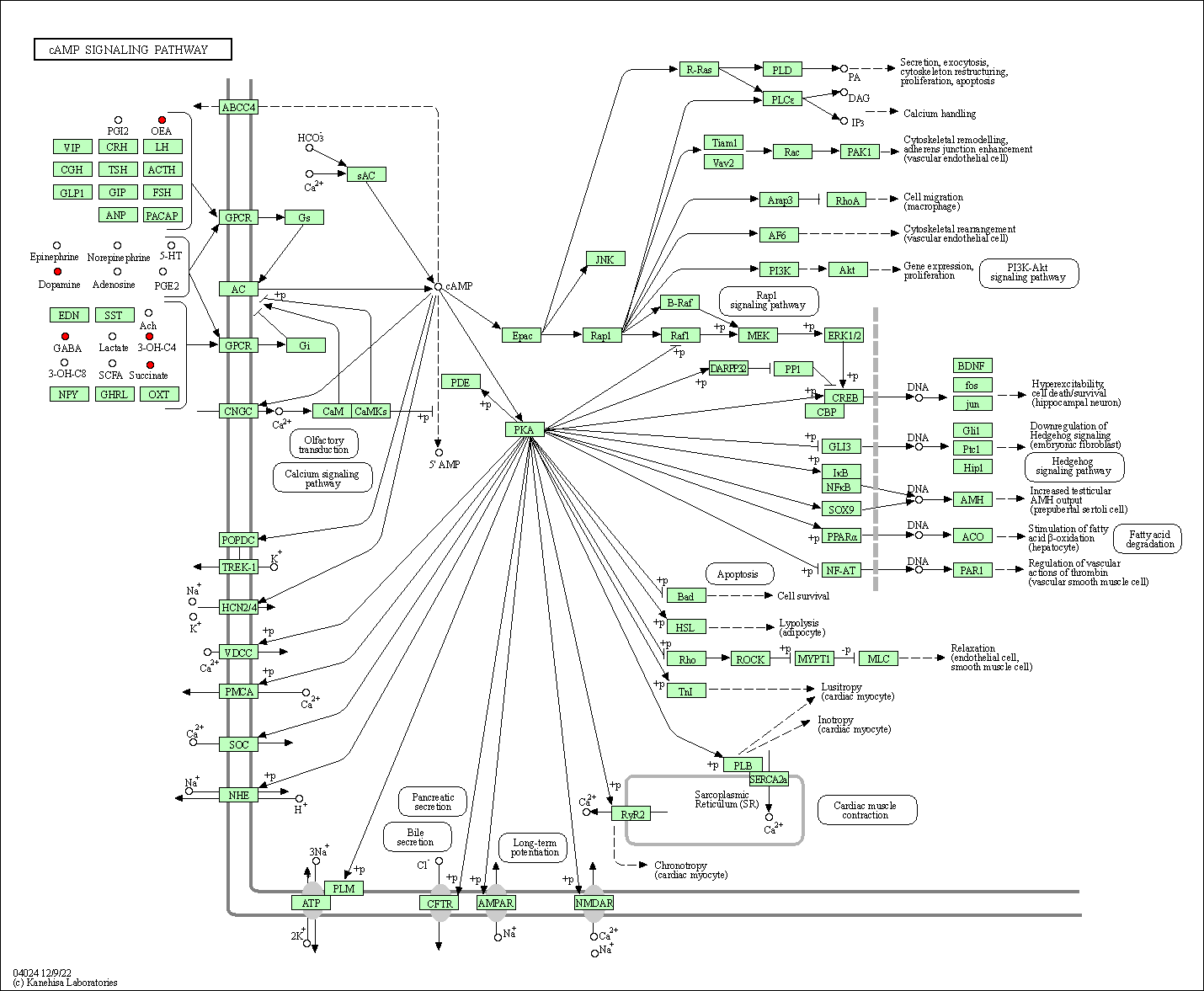


**Figure S3.** Schematic diagram of the cAMP pathway.

**Table S1.** Biomarkers tentatively identified in rat serum.

| Name | ID | mz | Rt (min) | Error (ppm) | Formula | Model_Mean | Control_Mean | P | VIP | Pos/Neg |
| --- | --- | --- | --- | --- | --- | --- | --- | --- | --- | --- |
| dTMP | M321T306 | 321.0398 | 306.3 | 29.59135 | C10H15N2O8P | 73780.3 | 4668232.33 | 5.3E-09 | 1.63003 | neg |
| 5,7-Dihydroxyflavone | M253T614 | 253.0501 | 614.1 | 0.737684 | C15H10O4 | 231501.15 | 4097250.98 | 1.15E-08 | 1.626184 | neg |
| Mitragynine | M397T709 | 397.2041 | 708.5 | 23.11517 | C23H30N2O4 | 49092.37 | 1369456.72 | 1.8E-08 | 1.622505 | neg |
| Prostaglandin E2 | M353T732 | 353.2465 | 731.6 | 5.479535 | C20H32O5 | 713443.81 | 15914140.09 | 3.17E-07 | 1.910012 | pos |
| 6-Hydroxydaidzein | M270T522 | 270.0782 | 522.2 | 7.222884 | C15H10O5 | 3442677.66 | 13873732.9 | 2.06E-06 | 1.880947 | pos |
| Baicalein | M271T525 | 271.0598 | 524.6 | 1.018224 | C15H10O5 | 451528.56 | 3182222.87 | 2.4E-06 | 1.877516 | pos |
| Formononetin | M268T563 | 268.065 | 562.9 | 14.75078 | C16H12O4 | 5951761.16 | 15767064.68 | 1.38E-05 | 1.561832 | neg |
| (5-L-Glutamyl)-L-glutamate | M277T139 | 277.1028 | 139.1 | 0.99602 | C10H16N2O7 | 1066611.66 | 2979192.32 | 5.8E-05 | 1.809061 | pos |
| L-Aspartic acid | M132T80 | 132.0289 | 80.2 | 1.196942 | C4H7NO4 | 53925429.9 | 123459110.3 | 7.66E-05 | 1.520417 | neg |
| 5-Methoxyindoleacetate | M204T445 | 204.066 | 444.7 | 2.940225 | C11H11NO3 | 955575.19 | 5425633.64 | 0.000113 | 1.508051 | neg |
| Baicalin | M447T524 | 447.0882 | 523.8 | 8.8931 | C21H18O11 | 1972652.67 | 8174505.82 | 0.000114 | 1.805407 | pos |
| N-Acetyl-D-glucosamine | M221T688 | 221.1538 | 687.8 | 1.798241 | C8H15NO6 | 6490449.4 | 8461965.65 | 0.000277 | 1.498742 | neg |
| Indoleacetaldehyde | M159T313 | 159.0678 | 313.2 | 1.279734 | C10H9NO | 1783400.33 | 3472022.51 | 0.000277 | 1.748601 | pos |
| 6-Hydroxyhexanoic acid | M131T224_2 | 131.0699 | 223.9 | 10.86443 | C6H12O3 | 8557176.74 | 22588229.66 | 0.000344 | 1.484307 | neg |
| 5-Methyl-2'-deoxycytidine | M240T300 | 240.0983 | 299.5 | 3.015432 | C10H15N3O4 | 2222211.93 | 3834444.58 | 0.000801 | 1.419459 | neg |
| Indoleglycerol phosphate | M288T88 | 288.0727 | 88.3 | 6.987402 | C11H14NO6P | 19187376.6 | 34848090.27 | 0.000925 | 1.70415 | pos |
| (Z)-4-Hydroxy-6-dodecenoic acid lactone | M179T404 | 179.0705 | 404.5 | 1.387918 | C6H10O6 | 1400359.22 | 2928469.87 | 0.000987 | 1.66959 | pos |
| Thymine | M127T338 | 127.0509 | 338.3 | 1.401401 | C5H6N2O2 | 7811896.35 | 13109943.48 | 0.001004 | 1.688889 | pos |
| O-Ureido-L-serine | M163T314 | 163.0627 | 313.9 | 20.85088 | C4H9N3O4 | 1216154.84 | 2196989.21 | 0.00124 | 1.665986 | pos |
| Chavicol | M135T709 | 135.0803 | 709 | 0.277793 | C9H10O | 747557.6 | 2835366.64 | 0.001967 | 1.621014 | pos |
| Kynurenic acid | M190T415 | 190.0493 | 415.4 | 3.030793 | C10H7NO3 | 5505205.91 | 10213250.03 | 0.002106 | 1.622841 | pos |
| Maltol | M127T356 | 127.0401 | 355.8 | 5.943134 | C6H6O3 | 11623996.9 | 54259517.42 | 0.002257 | 1.652068 | pos |
| Salicyluric acid | M194T378 | 194.0449 | 378.2 | 5.153446 | C9H9NO4 | 625103.26 | 1387680.38 | 0.002369 | 1.366522 | neg |
| L-Histidine | M156T86 | 156.0765 | 85.6 | 1.768364 | C6H9N3O2 | 10450175.9 | 23468388.67 | 0.002695 | 1.637607 | pos |
| 11Z-Eicosenoic acid | M293T967 | 293.2829 | 967.2 | 10.68593 | C20H38O2 | 16834762.1 | 40136009.73 | 0.002961 | 1.632395 | pos |
| Antibiotic G-418 | M496T610 | 496.2673 | 610.1 | 14.3066 | C20H40N4O10 | 9102467.42 | 21561832.64 | 0.003096 | 1.57754 | pos |
| S-Allylcysteine | M162T415 | 162.0548 | 415.4 | 20.94902 | C6H11NO2S | 9335203.1 | 16667091.05 | 0.003639 | 1.574298 | pos |
| Spermidine | M144T61 | 143.9144 | 60.7 | 0.683589 | C7H19N3 | 6364043.48 | 8596018.4 | 0.003948 | 1.352016 | neg |
| all-trans-Retinoic acid | M299T908 | 299.2008 | 908.1 | 2.160867 | C20H28O2 | 7345914.57 | 51603092.31 | 0.004069 | 1.327525 | neg |
| 5-Hydroxytryptophan | M203T439 | 203.0811 | 439.1 | 18.38674 | C11H12N2O3 | 4610572.33 | 12469265.55 | 0.00441 | 1.713053 | pos |
| gamma-Glutamyl-beta-aminopropiononitrile | M199T708 | 199.1108 | 707.9 | 16.12307 | C8H13N3O3 | 282043.39 | 1272231.75 | 0.004802 | 1.540146 | pos |
| trans-Cinnamate | M147T647 | 146.9647 | 646.8 | 0.978126 | C9H8O2 | 2314794.78 | 7674958.49 | 0.005705 | 1.306189 | neg |
| 5-Hydroxymethyl-2-furancarboxaldehyde | M127T986 | 127.0394 | 985.9 | 0.221012 | C6H6O3 | 19191329.8 | 34933838.49 | 0.006271 | 1.54541 | pos |
| 4-Methylcatechol | M123T455 | 123.0438 | 455.3 | 10.7604 | C7H8O2 | 341165.18 | 1128386.3 | 0.006851 | 1.400771 | neg |
| 4-Hydroxy-2-quinolone | M162T484 | 162.0557 | 483.8 | 4.4676 | C9H7NO2 | 2802374.68 | 5180214.92 | 0.007064 | 1.511717 | pos |
| (R)-4-Hydroxymandelate | M169T148 | 169.0603 | 147.8 | 10.91885 | C8H8O4 | 1461191.98 | 2531990.15 | 0.007157 | 1.499534 | pos |
| 4-Methoxy-2,2'-bipyrrole-5-carbaldehyde | M173T439 | 173.0708 | 439.1 | 23.30838 | C10H10N2O2 | 2519290.96 | 6529099.29 | 0.007724 | 1.653452 | pos |
| Docosatetraenoyl Ethanolamide | M376T939 | 376.3163 | 939 | 4.034159 | C24H41NO2 | 9282290.65 | 24867985.31 | 0.007774 | 1.493144 | pos |
| D-Glucopyranoside | M161T985 | 161.0432 | 984.6 | 11.23909 | C6H12O6 | 4612315.48 | 33496355.84 | 0.007827 | 1.267137 | neg |
| 9,10-Epoxyoctadecenoic acid | M295T880 | 295.2276 | 879.6 | 0.758737 | C18H32O3 | 80838117.9 | 136626080 | 0.008073 | 1.268973 | neg |
| L-Fucose | M164T377 | 164.0695 | 376.5 | 6.094978 | C6H12O5 | 3431513.92 | 4670531.67 | 0.008851 | 1.487274 | pos |
| Isoliquiritigenin | M257T513 | 257.0802 | 513.2 | 2.62953 | C15H12O4 | 438156.09 | 1689566.97 | 0.009319 | 1.636292 | pos |
| Indole-3-acetaldehyde oxime | M175T439_2 | 175.0864 | 438.9 | 1.005218 | C10H10N2O | 4131434.32 | 10399383.78 | 0.011938 | 1.613689 | pos |
| Glutaric acid | M132T502 | 132.0442 | 501.9 | 14.38912 | C5H8O4 | 1010760.09 | 2682522.07 | 0.012287 | 1.582622 | pos |
| Indole-3-carboxylic acid | M162T517 | 162.0558 | 517 | 5.084668 | C9H7NO2 | 2804034.65 | 4503130.88 | 0.013866 | 1.411497 | pos |
| Dimethylglycine | M102T80 | 102.0545 | 80.2 | 3.571661 | C4H9NO2 | 19741824.8 | 27710072.85 | 0.014254 | 1.185124 | neg |
| cis-4-Hydroxy-D-proline | M132T987_2 | 131.974 | 987.2 | 2.651853 | C5H9NO3 | 32381702.6 | 44365341.73 | 0.016308 | 1.416596 | pos |
| Quinaldic acid | M174T502 | 174.0544 | 501.9 | 3.295433 | C10H7NO2 | 4482516.37 | 12692393.71 | 0.016931 | 1.568418 | pos |
| N-Acetyl-L-aspartic acid | M158T502 | 158.0446 | 501.9 | 24.89171 | C6H9NO5 | 480573.52 | 1618398.72 | 0.018124 | 1.529921 | pos |
| Gemfibrozil | M249T765 | 249.1489 | 764.5 | 2.809565 | C15H22O3 | 47894302.2 | 70421798.41 | 0.019879 | 1.159705 | neg |
| 3-Dehydroshikimate | M172T34 | 171.9915 | 34.1 | 8.890747 | C7H8O5 | 11718581.3 | 17326079.12 | 0.020959 | 1.509164 | pos |
| 24-Methylenecycloartanol | M423T874 | 423.3965 | 873.6 | 5.040193 | C31H52O | 13328270.8 | 49183829.73 | 0.021064 | 1.374309 | pos |
| Nicotine | M161T981 | 160.9341 | 981.3 | 1.995455 | C10H14N2 | 7268087.54 | 9234361.03 | 0.021269 | 1.150764 | neg |
| Stigmastane | M400T894 | 400.4013 | 894.1 | 13.98577 | C29H52 | 39121281.4 | 79342199.73 | 0.023662 | 1.403845 | pos |
| Progesterone | M314T664 | 314.2323 | 664.4 | 24.50416 | C21H30O2 | 315424.57 | 1309252.95 | 0.029623 | 1.301104 | pos |
| Xanthoxin | M251T712 | 251.1634 | 711.7 | 2.317916 | C15H22O3 | 1568107.23 | 5333729.77 | 0.031403 | 1.289631 | pos |
| 1,2,3-Trihydroxybenzene | M127T254 | 127.0375 | 253.8 | 11.61862 | C6H6O3 | 17918514.3 | 40920099.6 | 0.032748 | 1.329695 | pos |
| Tetrahydropteridine | M136T335 | 136.0763 | 334.6 | 10.28835 | C6H8N4 | 5648163.06 | 9159516.3 | 0.033425 | 1.425732 | pos |
| Anserine | M241T86 | 241.1293 | 85.6 | 0.729899 | C10H16N4O3 | 6224298.41 | 11270714.96 | 0.034773 | 1.30821 | pos |
| Imidazol-5-yl-pyruvate | M155T474_3 | 155.0451 | 474.3 | 0.154794 | C6H6N2O3 | 1343807.09 | 1765542.66 | 0.03593 | 1.280803 | pos |
| Pimelic acid | M160T139 | 159.9687 | 138.6 | 2.697213 | C7H12O4 | 10198606.8 | 23627301.88 | 0.040054 | 1.278255 | pos |
| Xanthurenic acid | M206T392 | 206.0448 | 391.9 | 0.003442 | C10H7NO4 | 1662676.36 | 3229704.63 | 0.04173 | 1.383979 | pos |
| 4-(2-Aminophenyl)-2,4-dioxobutanoic acid | M208T90 | 208.06 | 89.7 | 2.287796 | C10H9NO4 | 2568986.6 | 5245450.73 | 0.042124 | 1.349538 | pos |
| Isovaleric acid | M102T35 | 102.1278 | 35.5 | 1.121377 | C5H10O2 | 8769200.33 | 11378779.08 | 0.049604 | 1.246947 | pos |
| 2-Pyrocatechuic acid | M154T474 | 154.0406 | 474.3 | 7.254771 | C7H6O4 | 14580483.2 | 18472061.33 | 0.049777 | 1.208583 | pos |
| 5'-O-beta-D-Glucosylpyridoxine | M332T154 | 332.1329 | 153.9 | 2.142663 | C14H21NO8 | 1489850.61 | 242175.72 | 5.45E-06 | 1.880928 | pos |
| Deoxycytidine | M226T154 | 226.0825 | 153.6 | 3.538531 | C9H13N3O4 | 9987845.8 | 1632972.55 | 6.45E-05 | 1.53686 | neg |
| 1-palmitoyl-dihydroxyacetone-phosphate | M407T782 | 407.2182 | 781.8 | 5.461416 | C19H37O7P | 4693950.16 | 1961023.21 | 0.000183 | 1.519268 | neg |
| Cortisol | M363T655 | 363.2152 | 654.7 | 3.788388 | C21H30O5 | 5907363.34 | 181811.64 | 0.000261 | 1.892159 | pos |
| Beta-Glycerophosphoric acid | M171T78 | 171.0053 | 78.4 | 6.424585 | C3H9O6P | 35722773.3 | 12902578.87 | 0.000304 | 1.506772 | neg |
| Secoisolariciresinol | M362T499 | 362.1639 | 499.1 | 24.85063 | C20H26O6 | 3210970.93 | 36515.24 | 0.00058 | 1.567057 | neg |
| 5-Methyl-2-furancarboxaldehyde | M110T319 | 110.0352 | 318.5 | 14.5408 | C6H6O2 | 2133952.79 | 1055964.37 | 0.00085 | 1.724039 | pos |
| Phenylacetylglycine | M194T496 | 194.0804 | 495.8 | 3.963368 | C10H11NO3 | 6690025.35 | 3186197.42 | 0.000996 | 1.700042 | pos |
| Phenylacetylglutamine | M263T365 | 263.1038 | 364.8 | 0.288859 | C13H16N2O4 | 3981191.82 | 1549147.89 | 0.001146 | 1.414907 | neg |
| Dolichotheline | M196T564 | 196.1445 | 563.8 | 0.122359 | C10H17N3O | 2231015.53 | 275215.91 | 0.001396 | 1.815822 | pos |
| (4Z,7Z,10Z,13Z,16Z,19Z)-Docosahexaenoic acid ethyl ester | M357T773 | 357.2778 | 772.6 | 2.731768 | C24H36O2 | 199710173 | 104946690.6 | 0.001798 | 1.634277 | pos |
| Trigonelline | M137T284 | 137.045 | 284.5 | 12.84658 | C7H7NO2 | 6201311.6 | 1103868.29 | 0.004024 | 1.724112 | pos |
| 4-Guanidinobutanoic acid | M146T143 | 146.0921 | 143.4 | 1.889219 | C5H11N3O2 | 6880199.46 | 3156462.42 | 0.004774 | 1.561742 | pos |
| Adenosine | M268T252 | 268.1039 | 252 | 0.379595 | C10H13N5O4 | 3442158.39 | 1520475.98 | 0.006225 | 1.558484 | pos |
| S-Adenosylhomocysteine | M383T307 | 383.1137 | 307.3 | 1.053393 | C14H20N6O5S | 1437635.34 | 791572.79 | 0.006776 | 1.301538 | neg |
| Xanthosine | M283T148_2 | 283.0674 | 147.7 | 3.617513 | C10H12N4O6 | 2411712.42 | 784495.77 | 0.007662 | 1.273972 | neg |
| DL-Glycerol 1-phosphate | M171T375 | 171.0109 | 374.8 | 26.17377 | C3H9O6P | 8006132.77 | 5846726.86 | 0.00857 | 1.296593 | neg |
| N-Acetyl-L-phenylalanine | M206T381 | 206.0812 | 381.2 | 1.383092 | C11H13NO3 | 11179879.9 | 4361857.78 | 0.011055 | 1.271617 | neg |
| L-Homophenylalanine | M180T375 | 180.1015 | 374.9 | 1.031175 | C10H13NO2 | 3723262.25 | 1910767.16 | 0.011497 | 1.614616 | pos |
| Gluconic acid | M195T81 | 195.0498 | 81.3 | 2.162913 | C6H12O7 | 1806800005 | 860015025.4 | 0.011622 | 1.356455 | neg |
| Hydroxykynurenine | M224T599 | 224.1276 | 599.3 | 2.373707 | C10H12N2O4 | 30172953.9 | 13640694.92 | 0.011885 | 1.45257 | pos |
| Guanine | M152T330 | 152.0566 | 330.1 | 0.001792 | C5H5N5O | 4892820.09 | 3267513.7 | 0.012921 | 1.478663 | pos |
| Tyrosol | M121T375 | 121.0649 | 374.8 | 1.161265 | C8H10O2 | 2778470.85 | 2011840.23 | 0.013603 | 1.472361 | pos |
| N2-gamma-Glutamylglutamine | M274T80 | 274.1041 | 80.4 | 1.182033 | C10H17N3O6 | 92997284.7 | 28867711.77 | 0.014068 | 1.241113 | neg |
| 1H-Indole-3-acetamide | M174T379 | 174.0549 | 379.3 | 1.32145 | C10H10N2O | 2797701.01 | 593588.51 | 0.014309 | 1.239593 | neg |
| N-Acetyl-D-tryptophan | M247T378 | 247.1282 | 378.1 | 2.374129 | C13H14N2O3 | 7241970.26 | 5503705.19 | 0.014648 | 1.473674 | pos |
| N-Formyl-L-glutamic acid | M176T495 | 176.0698 | 495.2 | 3.441877 | C6H9NO5 | 3534326.1 | 1044596.7 | 0.014729 | 1.446776 | pos |
| 15-Deoxy-d-12,14-PGJ2 | M315T537 | 315.1957 | 536.8 | 2.614249 | C20H28O3 | 3043103.55 | 2275802.57 | 0.017735 | 1.212764 | neg |
| 2-Phenylethyl acetate | M165T703 | 165.0908 | 703.4 | 1.06608 | C10H12O2 | 1958345.06 | 572157.42 | 0.018019 | 1.568312 | pos |
| L(-)-Carnitine | M162T218 | 162.1122 | 217.8 | 1.660887 | C7H15NO3 | 3487351.25 | 1996638.08 | 0.018471 | 1.428642 | pos |
| Cytidine | M242T152 | 242.0769 | 151.8 | 4.45762 | C9H13N3O5 | 12998879.4 | 7218734.81 | 0.020684 | 1.1809 | neg |
| L-Threonine | M118T90 | 118.0499 | 90.3 | 1.209617 | C4H9NO3 | 70825093.4 | 47037363.78 | 0.025515 | 1.142785 | neg |
| (S)-2,3-Epoxysqualene | M409T894 | 409.3825 | 893.7 | 9.121054 | C30H50O | 52999332.2 | 12166161.34 | 0.027989 | 1.360914 | pos |
| Cyclizine | M266T629 | 266.1854 | 628.8 | 26.67314 | C18H22N2 | 6390374.59 | 1144493.62 | 0.029926 | 1.462274 | pos |
| beta-D-Fructose 6-phosphate | M259T77 | 259.02 | 77 | 9.358351 | C6H13O9P | 11521144.7 | 7781948.22 | 0.031663 | 1.125276 | neg |
| L-Tyrosine | M180T153 | 180.0642 | 153.4 | 8.190555 | C9H11NO3 | 76738638.6 | 56663964.69 | 0.03329 | 1.130003 | neg |
| Threonic acid | M135T82 | 135.028 | 81.6 | 14.07116 | C4H8O5 | 432996897 | 328248362.6 | 0.033439 | 1.080438 | neg |
| 25-Hydroxycholesterol | M402T417 | 402.2121 | 417.3 | 1.485212 | C27H46O2 | 8414698.45 | 4499125.31 | 0.036655 | 1.283206 | pos |
| Dodecanedioic acid | M211T494 | 211.1334 | 493.9 | 0.047363 | C12H22O4 | 4718470.29 | 3052039.24 | 0.037522 | 1.085646 | neg |
| 2-Keto-6-acetamidocaproate | M170T367 | 170.0806 | 366.7 | 20.77838 | C8H13NO4 | 3698781.26 | 2518387.34 | 0.038196 | 1.304324 | pos |
| 5-Aminopentanoic acid | M116T932 | 115.919 | 932 | 5.188454 | C5H11NO2 | 224471726 | 180229922.2 | 0.041511 | 1.062637 | neg |
| 3-Methylthiopropionic acid | M119T154 | 119.0485 | 154.3 | 0.513204 | C4H8O2S | 6195437.88 | 3413083.5 | 0.048119 | 1.159066 | neg |
| L-Lysine | M145T93 | 145.097 | 93.3 | 1.367956 | C6H14N2O2 | 53107513.6 | 38362857.25 | 0.048628 | 1.078623 | neg |

Hits, the total number of substances in the target metabolic pathway; Up_hits, the number of up-regulated differential metabolites in the target metabolic pathway; Down_hits, the number of down-regulated differential metabolites in the target metabolic pathway.

**Table S2.** KEGG Pathway enrichment analysis of serum metabolites.

| Pathway_id | Pathway_name | Total | Hits | Up_hits | Down_hits | P | Impact |
| --- | --- | --- | --- | --- | --- | --- | --- |
| rno04974 | Protein digestion and absorption | 47 | 6 | 3 | 3 | 0.003765 | 0.1277 |
| rno02010 | ABC transporters | 138 | 10 | 6 | 4 | 0.01333 | 0.0725 |
| rno05200 | Pathways in cancer | 31 | 4 | 1 | 3 | 0.017023 | 0.1212 |
| rno00340 | Histidine metabolism+B5:O5 | 47 | 5 | 1 | 4 | 0.017278 | 0.152 |
| rno00380 | Tryptophan metabolism | 83 | 7 | 1 | 6 | 0.017366 | 0.0634 |
| rno00410 | beta-Alanine metabolism | 32 | 4 | 0 | 4 | 0.018978 | 0.1724 |
| rno00400 | Phenylalanine, tyrosine and tryptophan biosynthesis | 34 | 4 | 2 | 2 | 0.023293 | 0.1316 |
| rno00970 | Aminoacyl-tRNA biosynthesis | 52 | 5 | 3 | 2 | 0.025763 | 0.0893 |
| rno05034 | Alcoholism | 10 | 2 | 2 | 0 | 0.039897 | 0.1429 |
| rno00360 | Phenylalanine metabolism | 60 | 5 | 4 | 1 | 0.044178 | 0.0714 |
| rno04917 | Prolactin signaling pathway | 11 | 2 | 1 | 1 | 0.047745 | 0.1053 |
| rno05215 | Prostate cancer | 11 | 2 | 1 | 1 | 0.047745 | 0.1538 |
| rno04927 | Cortisol synthesis and secretion | 12 | 2 | 1 | 1 | 0.056099 | 0.1667 |
| rno04672 | Intestinal immune network for IgA production | 2 | 1 | 0 | 1 | 0.064106 | 0.5 |
| rno04934 | Cushing syndrome | 13 | 2 | 1 | 1 | 0.064921 | 0.1579 |
| rno04923 | Regulation of lipolysis in adipocytes | 14 | 2 | 1 | 1 | 0.074172 | 0.0714 |
| rno00310 | Lysine degradation | 50 | 4 | 3 | 1 | 0.078135 | 0.0455 |
| rno04080 | Neuroactive ligand-receptor interaction | 52 | 4 | 2 | 2 | 0.087501 | 0.0769 |
| rno05165 | Human papillomavirus infection | 3 | 1 | 0 | 1 | 0.094616 | 0.3333 |
| rno05222 | Small cell lung cancer | 3 | 1 | 0 | 1 | 0.094616 | 0.3333 |
| rno05224 | Breast cancer | 3 | 1 | 0 | 1 | 0.094616 | 0.3333 |
| rno05323 | Rheumatoid arthritis | 3 | 1 | 0 | 1 | 0.094616 | 0.3333 |
| rno04924 | Renin secretion | 17 | 2 | 1 | 1 | 0.104136 | 0.0741 |
| rno05230 | Central carbon metabolism in cancer | 37 | 3 | 1 | 2 | 0.117767 | 0.0566 |
| rno04114 | Oocyte meiosis | 4 | 1 | 0 | 1 | 0.124139 | 0.25 |
| rno04659 | Th17 cell differentiation | 4 | 1 | 0 | 1 | 0.124139 | 0.25 |
| rno04914 | Progesterone-mediated oocyte maturation | 4 | 1 | 0 | 1 | 0.124139 | 0.25 |
| rno05226 | Gastric cancer | 4 | 1 | 0 | 1 | 0.124139 | 0.25 |
| rno05012 | Parkinson disease | 21 | 2 | 2 | 0 | 0.148065 | 0.0606 |
| rno00240 | Pyrimidine metabolism | 65 | 4 | 2 | 2 | 0.160135 | 0.1151 |
| rno04916 | Melanogenesis | 6 | 1 | 1 | 0 | 0.180354 | 0.1667 |
| rno05140 | Leishmaniasis | 6 | 1 | 0 | 1 | 0.180354 | 0.1667 |
| rno05163 | Human cytomegalovirus infection | 6 | 1 | 0 | 1 | 0.180354 | 0.1667 |
| rno05223 | Non-small cell lung cancer | 6 | 1 | 0 | 1 | 0.180354 | 0.1667 |
| rno04024 | cAMP signaling pathway | 25 | 2 | 1 | 1 | 0.19496 | 0.0741 |
| rno05030 | Cocaine addiction | 7 | 1 | 1 | 0 | 0.207106 | 0.0909 |
| rno05033 | Nicotine addiction | 7 | 1 | 0 | 1 | 0.207106 | 0.1429 |
| rno00260 | Glycine, serine and threonine metabolism | 50 | 3 | 1 | 2 | 0.221694 | 0.0528 |
| rno00780 | Biotin metabolism | 28 | 2 | 1 | 1 | 0.231225 | 0.0938 |
| rno00250 | Alanine, aspartate and glutamate metabolism | 28 | 2 | 0 | 2 | 0.231225 | 0.2107 |
| rno05032 | Morphine addiction | 8 | 1 | 1 | 0 | 0.232992 | 0.1 |
| rno05143 | African trypanosomiasis | 8 | 1 | 0 | 1 | 0.232992 | 0.125 |
| rno04960 | Aldosterone-regulated sodium reabsorption | 8 | 1 | 1 | 0 | 0.232992 | 0.2 |
| rno00330 | Arginine and proline metabolism | 78 | 4 | 2 | 2 | 0.248293 | 0.0637 |
| rno05031 | Amphetamine addiction | 9 | 1 | 1 | 0 | 0.258041 | 0.0769 |
| rno04022 | cGMP-PKG signaling pathway | 10 | 1 | 1 | 0 | 0.28228 | 0.0714 |
| rno00030 | Pentose phosphate pathway | 35 | 2 | 2 | 0 | 0.316721 | 0.1641 |
| rno04728 | Dopaminergic synapse | 12 | 1 | 1 | 0 | 0.328427 | 0.0455 |
| rno04625 | C-type lectin receptor signaling pathway | 12 | 1 | 0 | 1 | 0.328427 | 0.0833 |
| rno04921 | Oxytocin signaling pathway | 12 | 1 | 0 | 1 | 0.328427 | 0.0833 |
| rno00270 | Cysteine and methionine metabolism | 63 | 3 | 2 | 1 | 0.337786 | 0.0313 |
| rno05146 | Amoebiasis | 13 | 1 | 0 | 1 | 0.350386 | 0.0769 |
| rno04071 | Sphingolipid signaling pathway | 15 | 1 | 1 | 0 | 0.392191 | 0.0345 |
| rno04270 | Vascular smooth muscle contraction | 16 | 1 | 1 | 0 | 0.412083 | 0.0625 |
| rno00232 | Caffeine metabolism | 22 | 1 | 1 | 0 | 0.518589 | 0.0294 |
| rno04925 | Aldosterone synthesis and secretion | 22 | 1 | 0 | 1 | 0.518589 | 0.1 |
| rno00051 | Fructose and mannose metabolism | 54 | 2 | 1 | 1 | 0.530588 | 0.119 |
| rno04714 | Thermogenesis | 23 | 1 | 0 | 1 | 0.534378 | 0.0256 |
| rno00290 | Valine, leucine and isoleucine biosynthesis | 23 | 1 | 1 | 0 | 0.534378 | 0.0645 |
| rno00220 | Arginine biosynthesis | 23 | 1 | 0 | 1 | 0.534378 | 0.0943 |
| rno00760 | Nicotinate and nicotinamide metabolism | 55 | 2 | 1 | 1 | 0.540584 | 0.0233 |
| rno04913 | Ovarian steroidogenesis | 24 | 1 | 0 | 1 | 0.549654 | 0.0455 |
| rno00830 | Retinol metabolism | 25 | 1 | 0 | 1 | 0.564433 | 0.0725 |
| rno00100 | Steroid biosynthesis | 58 | 2 | 1 | 1 | 0.569693 | 0.0299 |
| rno00230 | Purine metabolism | 95 | 3 | 3 | 0 | 0.6051 | 0.0665 |
| rno00591 | Linoleic acid metabolism | 28 | 1 | 0 | 1 | 0.605947 | 0.0263 |
| rno04978 | Mineral absorption | 29 | 1 | 1 | 0 | 0.618894 | 0.0286 |
| rno04976 | Bile secretion | 97 | 3 | 1 | 2 | 0.619401 | 0.0275 |
| rno00770 | Pantothenate and CoA biosynthesis | 30 | 1 | 0 | 1 | 0.63142 | 0.0357 |
| rno00730 | Thiamine metabolism | 31 | 1 | 1 | 0 | 0.643538 | 0.0222 |
| rno00010 | Glycolysis / Gluconeogenesis | 31 | 1 | 1 | 0 | 0.643538 | 0.0705 |
| rno00920 | Sulfur metabolism | 33 | 1 | 1 | 0 | 0.666601 | 0.0182 |
| rno04750 | Inflammatory mediator regulation of TRP channels | 35 | 1 | 0 | 1 | 0.688186 | 0.0256 |
| rno00590 | Arachidonic acid metabolism | 75 | 2 | 1 | 1 | 0.709424 | 0.0203 |
| rno00480 | Glutathione metabolism | 38 | 1 | 0 | 1 | 0.717992 | 0.028 |
| rno00350 | Tyrosine metabolism | 78 | 2 | 2 | 0 | 0.729773 | 0.048 |
| rno04726 | Serotonergic synapse | 42 | 1 | 0 | 1 | 0.753387 | 0.0161 |
| rno00120 | Primary bile acid biosynthesis | 47 | 1 | 1 | 0 | 0.791498 | 0.0098 |
| rno00130 | Ubiquinone and other terpenoid-quinone biosynthesis | 92 | 2 | 1 | 1 | 0.809508 | 0.0187 |
| rno00071 | Fatty acid degradation | 50 | 1 | 0 | 1 | 0.8115 | 0.003 |
| rno00053 | Ascorbate and aldarate metabolism | 50 | 1 | 1 | 0 | 0.8115 | 0.0065 |
| rno00140 | Steroid hormone biosynthesis | 99 | 2 | 1 | 1 | 0.841009 | 0.0558 |
| rno00520 | Amino sugar and nucleotide sugar metabolism | 108 | 2 | 1 | 1 | 0.874616 | 0.0517 |
| rno01040 | Biosynthesis of unsaturated fatty acids | 74 | 1 | 0 | 1 | 0.916162 | 0.0104 |
| rno00524 | Neomycin, kanamycin and gentamicin biosynthesis | 81 | 1 | 0 | 1 | 0.933883 | 0.012 |
| rno00980 | Metabolism of xenobiotics by cytochrome P450 | 121 | 1 | 0 | 1 | 0.983149 | 0.004 |
| rno00860 | Porphyrin and chlorophyll metabolism | 142 | 1 | 1 | 0 | 0.991836 | 0.002 |

Hits, the total number of substances in the target metabolic pathway; Up_hits, the number of up-regulated differential metabolites in the target metabolic pathway; Down_hits, the number of down-regulated differential metabolites in the target metabolic pathway.

**Table S3.** Biomarkers tentatively identified in rat soft palate tissue.

| Name | ID | mz | Rt (min) | Error (ppm) | Formula | Model_Mean | | Control_Mean | P | VIP | Pos/Neg |
| --- | --- | --- | --- | --- | --- | --- | --- | --- | --- | --- | --- |
| NADH | M664T126_2 | 664.1225 | 126.2 | 7.492594 | C21H29N7O14P2 | 40187017.8 | 1657843.66 | | 1.8E-08 | 1.756967 | neg |
| Nicotinamide ribotide | M335T102_1 | 335.0652 | 102.2 | 2.644648 | C11H15N2O8P | 41597781.5 | 226294.02 | | 3.04E-07 | 1.753913 | pos |
| D-Xylitol | M151T91 | 151.0615 | 90.8 | 1.82707 | C5H12O5 | 21967536.1 | 4302303.82 | | 5.93E-07 | 1.730677 | neg |
| N(omega)-Nitro-L-arginine methyl ester | M233T124 | 233.1127 | 124.2 | 0.68616 | C7H15N5O4 | 92766738.9 | 19848460.4 | | 7.1E-07 | 1.745027 | pos |
| 5-KETE | M301T772 | 301.2185 | 771.9 | 21.35991 | C20H30O3 | 35611097.4 | 268825.31 | | 8.15E-07 | 1.73484 | pos |
| N6-Acetyl-L-lysine | M189T124 | 189.123 | 124.2 | 1.988124 | C8H16N2O3 | 129110312 | 50478974.1 | | 8.71E-07 | 1.746705 | pos |
| Chenodeoxycholic acid | M391T801 | 391.2853 | 800.8 | 0.316904 | C24H40O4 | 133109855 | 1479854.5 | | 1.02E-06 | 1.733544 | neg |
| Riboflavin | M375T448_1 | 375.1313 | 447.6 | 0.735742 | C17H20N4O6 | 6125524.78 | 1839660.86 | | 3.62E-06 | 1.712546 | neg |
| Deoxycytidine | M228T125 | 228.0973 | 124.8 | 0.698869 | C9H13N3O4 | 193801353 | 105954335 | | 4.05E-06 | 1.731824 | pos |
| Aspartame | M277T501 | 277.1188 | 501.1 | 16.72207 | C14H18N2O5 | 5928222.5 | 1959203.42 | | 6.54E-06 | 1.719723 | pos |
| N-methyl-L-glutamic Acid | M160T113 | 160.0618 | 113.4 | 1.675403 | C6H11NO4 | 15378962.2 | 3644134.24 | | 6.76E-06 | 1.709816 | neg |
| beta-Alanyl-L-lysine | M217T324 | 217.1546 | 323.6 | 0.017451 | C9H19N3O3 | 335169821 | 26550499.9 | | 1E-05 | 1.70753 | pos |
| Gentisic acid | M155T975 | 154.99 | 974.9 | 0.116924 | C7H6O4 | 360869579 | 246897747 | | 1.56E-05 | 1.721296 | pos |
| 1-(3,4-Dihydroxyphenyl)-5-hydroxy-3-decanone | M281T360 | 281.1502 | 360 | 0.453835 | C16H24O4 | 46596862.8 | 16096856 | | 2.3E-05 | 1.696963 | pos |
| N-Acetyl-L-phenylalanine | M206T380 | 206.0822 | 379.5 | 0.116458 | C11H13NO3 | 35312647.9 | 10372015.8 | | 2.32E-05 | 1.685551 | neg |
| Pilocarpine | M209T423 | 209.1287 | 423.2 | 0.29722 | C11H16N2O2 | 9096687.35 | 2553732.64 | | 2.64E-05 | 1.684738 | pos |
| Glycerophosphocholine | M258T100 | 258.1096 | 100 | 0.587254 | C8H21NO6P | 129907632 | 59742243.5 | | 4.87E-05 | 1.672351 | pos |
| Dopamine | M136T151 | 136.0756 | 151 | 29.64529 | C8H11NO2 | 343634999 | 187990190 | | 6.44E-05 | 1.680304 | pos |
| Nicotianamine | M303T307_2 | 303.1463 | 306.9 | 10.88583 | C12H21N3O6 | 19129816.2 | 3235051.91 | | 9.45E-05 | 1.647075 | pos |
| Spermidine | M146T68 | 146.1654 | 67.8 | 0.553791 | C7H19N3 | 540246919 | 179846512 | | 0.00011 | 1.650422 | pos |
| Pelargonic acid | M158T766 | 158.1541 | 766.1 | 3.28894 | C9H18O2 | 109979791 | 85627935.7 | | 0.000124 | 1.663166 | pos |
| (-)-alpha-Narcotine | M413T501 | 413.136 | 500.7 | 27.83587 | C22H23NO7 | 2800182.97 | 40401.44 | | 0.000143 | 1.634176 | pos |
| gamma-Glutamylalanine | M219T147 | 219.0967 | 147.2 | 3.119532 | C7H11N2O5R | 28423652.3 | 11496466.9 | | 0.000145 | 1.62878 | pos |
| Anandamide | M348T913 | 348.2885 | 913.1 | 3.376511 | C22H37NO2 | 28498016.4 | 11693211.8 | | 0.000173 | 1.642975 | pos |
| Cytosine | M112T120 | 112.0503 | 120 | 2.463179 | C4H5N3O | 1148220095 | 674583871 | | 0.000203 | 1.642832 | pos |
| 4-Quinolinecarboxylic acid | M173T527 | 173.1188 | 526.5 | 10.38575 | C10H7NO2 | 1734484.8 | 876551.99 | | 0.000204 | 1.708139 | neg |
| Benzophenone | M183T817 | 183.0805 | 816.6 | 0.13109 | C13H10O | 40568885.6 | 31764127.7 | | 0.000211 | 1.637995 | pos |
| 2-Hydroxybutyric acid | M104T125 | 104.053 | 125.1 | 1.815007 | C4H8O3 | 175689949 | 98739668.4 | | 0.000217 | 1.639412 | pos |
| 5,6-Dihydro-5-fluorouracil | M133T125 | 133.0315 | 125.1 | 1.955697 | C4H5FN2O2 | 489983563 | 276906455 | | 0.00022 | 1.640372 | pos |
| myo-Inositol | M181T975 | 180.9899 | 975.2 | 2.114969 | C6H12O6 | 67848438 | 45720496.2 | | 0.000251 | 1.647747 | pos |
| S-(Hydroxymethyl)glutathione | M320T183 | 320.0907 | 183 | 11.66558 | C11H19N3O7S | 76690499.4 | 1179576.9 | | 0.000311 | 1.604459 | pos |
| Dimethylglycine | M104T89 | 104.0707 | 88.6 | 0.960885 | C4H9NO2 | 62772402.6 | 24586721 | | 0.000313 | 1.596098 | pos |
| Isotretinoin | M301T745 | 301.2153 | 745.2 | 3.008294 | C20H28O2 | 37103319 | 4956909.75 | | 0.000319 | 1.598643 | pos |
| Nebularine | M253T333 | 253.0933 | 332.8 | 0.489938 | C10H12N4O4 | 36266754.4 | 4457311.97 | | 0.000403 | 1.607108 | pos |
| 2-Ketobutyric acid | M103T89 | 103.039 | 89.1 | 0.232922 | C4H6O3 | 29924365.7 | 21987883.7 | | 0.000413 | 1.604999 | pos |
| 2-Hydroxyglutarate | M148T116 | 148.043 | 116.3 | 2.401324 | C5H8O5 | 121444840 | 23537330.6 | | 0.000482 | 1.586212 | pos |
| 5(S)-HpETE | M319T745 | 319.2262 | 745.2 | 1.237162 | C20H32O4 | 55257905.8 | 9706997.37 | | 0.000502 | 1.578186 | pos |
| Acetylcysteine | M163T715 | 163.039 | 714.6 | 0.476934 | C5H9NO3S | 6877196.45 | 5591290.02 | | 0.00065 | 1.597717 | pos |
| Phenylacetylglycine | M192T369 | 192.0671 | 368.5 | 2.478301 | C10H11NO3 | 101720461 | 33632986.3 | | 0.00076 | 1.56856 | neg |
| 2-Methoxy-17beta-estradiol | M302T736 | 302.1888 | 736.3 | 1.985514 | C19H26O3 | 9504480.69 | 3291864.32 | | 0.000798 | 1.594946 | pos |
| L-Prolinamide | M115T76 | 115.0867 | 75.5 | 1.07745 | C5H10N2O | 17413207.4 | 5168763.28 | | 0.00085 | 1.558723 | pos |
| Prostaglandin E1 | M337T772 | 337.2372 | 771.9 | 2.07569 | C20H34O5 | 37410631.5 | 1270649.49 | | 0.000939 | 1.67026 | pos |
| Glycylleucine | M189T299 | 189.1238 | 299.3 | 2.241918 | C8H16N2O3 | 234591992 | 98378976.1 | | 0.00095 | 1.568649 | pos |
| N-Acetylhistidine | M198T196 | 198.0878 | 195.7 | 2.524133 | C8H11N3O3 | 193287656 | 25358288 | | 0.001199 | 1.518797 | pos |
| gamma-Aminobutyric acid | M104T117 | 104.1071 | 116.9 | 2.725348 | C4H9NO2 | 75400075.8 | 40593264.6 | | 0.001225 | 1.517883 | pos |
| Acetyl adenylate | M388T67 | 388.0651 | 67.3 | 3.1541 | C12H16N5O8P | 41360907 | 12160020.4 | | 0.001259 | 1.5262 | neg |
| Hydroxylaminobenzene | M110T514 | 110.06 | 514.4 | 0.690532 | C6H7NO | 12107881.2 | 4033633.71 | | 0.001264 | 1.521651 | pos |
| Troxilin B3 | M353T693 | 353.2327 | 692.7 | 1.766538 | C20H34O5 | 66729812.4 | 5482115.83 | | 0.001422 | 1.52084 | neg |
| Chelirubine | M362T264 | 362.0866 | 263.8 | 2.816708 | C21H16NO5 | 9990020.98 | 2648934.28 | | 0.001601 | 1.529249 | pos |
| Nicotinamide riboside | M255T99 | 255.0969 | 99.1 | 1.140182 | C11H15N2O5 | 7994461.08 | 1761766.14 | | 0.001627 | 1.533327 | pos |
| 3-Hydroxyanthranilate | M153T319 | 153.0408 | 319.1 | 11.71646 | C7H7NO3 | 173932120 | 55556782.7 | | 0.001823 | 1.531517 | pos |
| Methyleugenol | M179T800 | 179.1067 | 799.8 | 0.009715 | C11H14O2 | 26650803 | 19542383.1 | | 0.001956 | 1.532497 | pos |
| N-Acetyl-L-glutamate 5-semialdehyde | M172T119 | 172.0619 | 119.1 | 2.185265 | C7H11NO4 | 40689013.2 | 12475823.9 | | 0.002016 | 1.508041 | neg |
| 13-L-Hydroperoxylinoleic acid | M293T862 | 293.2121 | 862.5 | 1.330095 | C18H32O4 | 144430401 | 26540919.5 | | 0.002032 | 1.487472 | neg |
| (4Z,7Z,10Z,13Z,16Z,19Z)-Docosahexaenoic acid ethyl ester | M357T773 | 357.2772 | 773.1 | 4.411141 | C24H36O2 | 219873992 | 36729581.7 | | 0.002185 | 1.529031 | pos |
| Gluconic acid | M195T81 | 195.0509 | 80.6 | 0.635731 | C6H12O7 | 979235752 | 484624589 | | 0.002809 | 1.463543 | neg |
| 3-(2-Hydroxyphenyl)propanoic acid | M167T829 | 167.0704 | 828.9 | 0.04612 | C9H10O3 | 30421239.8 | 23307870.2 | | 0.002847 | 1.501132 | pos |
| Cytidine | M244T120 | 244.093 | 120 | 0.384575 | C9H13N3O5 | 1864559385 | 1027343365 | | 0.003114 | 1.482216 | pos |
| Quinolinic acid | M167T72 | 167.013 | 72.2 | 0.523465 | C7H5NO4 | 51748818.8 | 24448061.1 | | 0.003147 | 1.589381 | pos |
| 2-Aminobenzoic acid | M136T434 | 136.0407 | 433.7 | 2.028805 | C7H7NO2 | 3235680.19 | 1534792.85 | | 0.003542 | 1.470405 | neg |
| Taurocyamine | M166T93_1 | 166.0299 | 93.4 | 4.071572 | C3H9N3O3S | 15601221.8 | 9263947.37 | | 0.003676 | 1.460297 | neg |
| Betaine | M118T90 | 118.0862 | 90.3 | 0.643598 | C5H11NO2 | 1707885943 | 687961278 | | 0.003843 | 1.443373 | pos |
| Xanthosine | M283T315 | 283.0689 | 315.2 | 1.681569 | C10H12N4O6 | 102241797 | 27113396.9 | | 0.003981 | 1.600846 | neg |
| m-Cresol | M109T559 | 109.1012 | 559.1 | 2.413519 | C7H8O | 4921268.77 | 2896761.86 | | 0.004044 | 1.45711 | pos |
| Octanal | M129T606 | 129.1276 | 606 | 1.548856 | C8H16O | 6270711.42 | 5020304.34 | | 0.004205 | 1.474447 | pos |
| 12-KETE | M319T837 | 319.2256 | 837.2 | 3.683915 | C20H30O3 | 40923181.4 | 16611642.3 | | 0.004404 | 1.437965 | pos |
| Saccharopine | M276T99 | 276.1192 | 98.9 | 0.014197 | C11H20N2O6 | 115075619 | 62571515.4 | | 0.004477 | 1.426707 | pos |
| Pyridoxine | M169T123 | 169.0131 | 123.4 | 18.37427 | C8H11NO3 | 11399875.7 | 1121432.28 | | 0.00482 | 1.427187 | neg |
| 2-Biphenylol | M171T102 | 171.0764 | 101.8 | 23.82561 | C12H10O | 70111894.8 | 11398042.1 | | 0.005246 | 1.558367 | pos |
| D-Mannose 1-phosphate | M259T73 | 259.0234 | 73.1 | 3.767999 | C6H13O9P | 476800229 | 268428987 | | 0.005386 | 1.526343 | neg |
| 2-Keto-6-acetamidocaproate | M188T107 | 188.0921 | 107.4 | 0.04459 | C8H13NO4 | 29339649.9 | 2731873.59 | | 0.005872 | 1.563715 | pos |
| Gemfibrozil | M249T759 | 249.1504 | 759.1 | 3.114585 | C15H22O3 | 35282801.1 | 12640573.6 | | 0.005996 | 1.419837 | neg |
| L-Tyrosine | M180T126 | 180.0671 | 126.4 | 2.643459 | C9H11NO3 | 391362564 | 248767401 | | 0.006137 | 1.424026 | neg |
| Methyl jasmonate | M205T888 | 205.16 | 887.8 | 3.763485 | C13H20O3 | 15776200.3 | 9666132.39 | | 0.006449 | 1.428637 | neg |
| dIMP | M331T111 | 331.0454 | 110.8 | 1.437869 | C10H13N4O7P | 5826221.45 | 727238.83 | | 0.006459 | 1.40344 | neg |
| Formylanthranilic acid | M164T347 | 164.0361 | 347 | 4.730666 | C8H7NO3 | 1968539.41 | 994862.78 | | 0.006922 | 1.534073 | neg |
| 3,4-Dihydro-2H-1-benzopyran-2-one | M149T340 | 149.0597 | 340.5 | 0.036871 | C9H8O2 | 183041457 | 109233042 | | 0.006986 | 1.564447 | pos |
| 2-Methylserine | M119T129 | 119.0494 | 128.9 | 0.18971 | C4H9NO3 | 64221914.2 | 36027567 | | 0.00714 | 1.556364 | pos |
| Uracil | M113T126 | 113.0344 | 126 | 1.557048 | C4H4N2O2 | 162812397 | 119409898 | | 0.007523 | 1.406835 | pos |
| 3,4-Dihydroxyphenylpropanoate | M165T129 | 165.0545 | 128.8 | 24.44041 | C9H10O4 | 1076889516 | 621177436 | | 0.007676 | 1.543783 | pos |
| 4-Hydroxycinnamic acid | M163T158 | 163.0407 | 157.5 | 4.146204 | C9H8O3 | 3602968.77 | 2147736.75 | | 0.008017 | 1.394152 | neg |
| D-synephrine | M168T341 | 168.0912 | 340.7 | 2.269837 | C9H13NO2 | 78114578.3 | 47505341.6 | | 0.008571 | 1.503506 | pos |
| 4-Hydroxybenzaldehyde | M123T203 | 123.0441 | 203.1 | 0.195052 | C7H6O2 | 59653985.6 | 17052889.5 | | 0.008667 | 1.471284 | pos |
| Baicalein | M271T729 | 271.2744 | 729.2 | 1.289307 | C15H10O5 | 6737778.12 | 741130.22 | | 0.009081 | 1.509231 | pos |
| Prostaglandin F2a | M353T604 | 353.2337 | 603.6 | 1.064451 | C20H34O5 | 8272498.4 | 4272945.96 | | 0.0092 | 1.38024 | neg |
| Phenylacetylglutamine | M265T462 | 265.1183 | 461.9 | 0.090526 | C13H16N2O4 | 1464819.54 | 213066.83 | | 0.009217 | 1.401436 | pos |
| Deoxyuridine | M228T750 | 228.1955 | 749.7 | 0.895414 | C9H12N2O5 | 331410308 | 245448748 | | 0.0097 | 1.385017 | pos |
| Cholesterol sulfate | M465T910_2 | 465.3093 | 909.6 | 10.47905 | C27H46O4S | 121022992 | 91174431.5 | | 0.009902 | 1.370771 | neg |
| Benzaldehyde | M107T341 | 107.0492 | 340.5 | 0.224196 | C7H6O | 49316733.6 | 29268955.1 | | 0.010049 | 1.518461 | pos |
| Adrenic acid | M331T898 | 331.2647 | 897.8 | 1.207494 | C22H36O2 | 4196751683 | 2830348036 | | 0.010929 | 1.353121 | neg |
| Aminoadipic acid | M160T96 | 160.0618 | 96.4 | 1.724334 | C6H11NO4 | 16147048.3 | 9756462.76 | | 0.012197 | 1.315217 | neg |
| Capsidiol | M219T682 | 219.1745 | 681.8 | 0.00034 | C15H24O2 | 4253785.36 | 2842906.03 | | 0.012293 | 1.363811 | pos |
| 14,15-DiHETrE | M337T782 | 337.2384 | 781.6 | 0 | C20H34O4 | 5665647.38 | 1603618.25 | | 0.013789 | 1.316889 | neg |
| Deoxyinosine | M251T302 | 251.0796 | 301.8 | 3.887213 | C10H12N4O4 | 20700273.3 | 3655199.22 | | 0.013799 | 1.32948 | neg |
| Glycocholic acid | M464T551 | 464.3101 | 551.1 | 15.55906 | C26H43NO6 | 7119417.25 | 1178006.9 | | 0.013825 | 1.32451 | neg |
| 12-Hydroxydodecanoic acid | M215T706 | 215.1658 | 705.8 | 2.677005 | C12H24O3 | 5555479.96 | 2808921.97 | | 0.014373 | 1.297212 | neg |
| S-Adenosylhomocysteine | M383T297 | 383.1134 | 297.5 | 1.836452 | C14H20N6O5S | 8094033.98 | 3889127.92 | | 0.014474 | 1.289267 | neg |
| Sphingosine | M300T782_1 | 300.2891 | 781.8 | 1.918152 | C18H37NO2 | 45498824.4 | 25209750.9 | | 0.015563 | 1.314762 | pos |
| Succinic acid | M117T73 | 117.0196 | 73.3 | 2.358579 | C4H6O4 | 689054246 | 15110814.7 | | 0.016149 | 1.280629 | neg |
| L-Asparagine | M133T85 | 133.061 | 85.2 | 0.378887 | C4H8N2O3 | 71861294.4 | 48824026 | | 0.016223 | 1.312333 | pos |
| Pantetheine | M277T419 | 277.1237 | 419.1 | 3.521905 | C11H22N2O4S | 5724925.85 | 2530889.08 | | 0.017146 | 1.262003 | neg |
| 10-Hydroxydecanoic acid | M187T586 | 187.134 | 585.9 | 0.406126 | C10H20O3 | 9596059.23 | 5502413.52 | | 0.017464 | 1.281602 | neg |
| cis-4-Hydroxy-D-proline | M132T58 | 131.9733 | 57.8 | 7.95597 | C5H9NO3 | 31618235.4 | 26378623.9 | | 0.017589 | 1.422877 | pos |
| Indole | M117T114 | 117.0559 | 113.6 | 16.2313 | C8H7N | 39482734 | 14614782.8 | | 0.01815 | 1.279943 | neg |
| 2-(Methylamino)benzoic acid | M150T511 | 150.0567 | 510.7 | 4.314849 | C8H9NO2 | 1465200.31 | 794741.13 | | 0.018599 | 1.267779 | neg |
| 9-cis-Retinoic acid | M301T837 | 301.2155 | 837.2 | 2.24424 | C20H28O2 | 23491998.8 | 5474213.98 | | 0.018785 | 1.266138 | pos |
| Ecdysone | M464T665_2 | 464.3028 | 665.2 | 5.324934 | C27H44O6 | 92967275.6 | 21592471.8 | | 0.019065 | 1.424805 | pos |
| Nonadecanoic acid | M297T869 | 297.2464 | 869.5 | 9.321892 | C19H38O2 | 92333433.8 | 42491719.2 | | 0.020104 | 1.271652 | neg |
| Chavicol | M135T748 | 135.0805 | 747.9 | 0.177672 | C9H10O | 7915623.28 | 6699449.09 | | 0.020256 | 1.289328 | pos |
| Labetalol | M327T792_1 | 327.1809 | 792.3 | 28.96257 | C19H24N2O3 | 8819118.57 | 3221146.52 | | 0.020324 | 1.246303 | neg |
| L-Valine | M117T187 | 116.9293 | 187 | 17.41049 | C5H11NO2 | 30166360.9 | 7284382.84 | | 0.020666 | 1.286936 | neg |
| N-Formyl-L-methionine | M176T182 | 176.0388 | 181.7 | 0.431723 | C6H11NO3S | 4862792.37 | 1675596.87 | | 0.021058 | 1.276987 | neg |
| 3-Epiecdysone | M464T727 | 464.2855 | 727.2 | 4.815657 | C27H44O6 | 4977519.85 | 1275182.61 | | 0.021198 | 1.297598 | pos |
| Dethiobiotin | M197T407 | 197.129 | 407.4 | 24.01473 | C10H18N2O3 | 5583625.92 | 2203831.85 | | 0.021446 | 1.235281 | pos |
| Adipate semialdehyde | M130T980 | 130.0499 | 980.2 | 1.332647 | C6H10O3 | 117270459 | 102619098 | | 0.021683 | 1.260059 | pos |
| Deoxycholic acid | M392T718_2 | 392.2884 | 718.1 | 1.317316 | C24H40O4 | 4704599.43 | 652023.25 | | 0.021882 | 1.390908 | neg |
| Thymine | M127T337 | 127.0505 | 336.6 | 1.294763 | C5H6N2O2 | 28654350 | 6181782.02 | | 0.023784 | 1.295676 | pos |
| Oxalacetic acid | M113T90 | 112.9861 | 89.7 | 12.47941 | C4H4O5 | 400410626 | 289670540 | | 0.024197 | 1.253962 | neg |
| Pipecolic acid | M130T128 | 130.05 | 127.5 | 20.27465 | C6H11NO2 | 381372017 | 292057060 | | 0.025034 | 1.239747 | pos |
| 21-Deoxycortisol | M347T842 | 347.2198 | 841.7 | 5.402918 | C21H30O4 | 10161941.7 | 1215214.93 | | 0.026182 | 1.240514 | pos |
| L-Serine | M106T85_1 | 106.05 | 85.2 | 1.16926 | C3H7NO3 | 121606237 | 93573057 | | 0.027023 | 1.237007 | pos |
| [8]-Shogaol | M275T761 | 275.1693 | 761.2 | 14.53651 | C17H24O3 | 19995909.4 | 3053752.99 | | 0.027691 | 1.207267 | neg |
| (R)-3-Hydroxybutyric acid | M103T129 | 103.0403 | 129.2 | 2.678564 | C4H8O3 | 16880296.3 | 4363171.33 | | 0.027697 | 1.220018 | neg |
| Kynurenic acid | M190T421 | 190.0499 | 420.5 | 0.087821 | C10H7NO3 | 102952598 | 29451952.3 | | 0.028958 | 1.21835 | pos |
| Quercetin | M283T306 | 283.0689 | 306.4 | 27.91105 | C15H10O7 | 57937457 | 12556216 | | 0.030029 | 1.202286 | neg |
| Citicoline | M489T90_2 | 489.1107 | 90.2 | 7.924586 | C14H26N4O11P2 | 16947839.6 | 9598869.91 | | 0.031552 | 1.339209 | pos |
| L-beta-Phenylalanine | M166T567 | 166.0843 | 566.6 | 11.89743 | C9H11NO2 | 16753065.3 | 7501239.93 | | 0.031596 | 1.21542 | pos |
| 12-Keto-leukotriene B4 | M333T576 | 333.2081 | 576 | 2.9291 | C20H30O4 | 955407.04 | 177499.18 | | 0.03313 | 1.210994 | neg |
| Thyroxine | M777T933_2 | 776.5789 | 932.7 | 10.03638 | C15H11I4NO4 | 27787408.9 | 8650387.99 | | 0.034111 | 1.207169 | pos |
| Oleoylethanolamide | M326T971 | 326.3053 | 971.4 | 0.232911 | C20H39NO2 | 40980473.1 | 28902747.1 | | 0.035029 | 1.193907 | pos |
| ATP | M506T67 | 505.9855 | 66.8 | 5.778788 | C10H16N5O13P3 | 2166183.92 | 940266.7 | | 0.036565 | 1.208177 | neg |
| 6-Methylmercaptopurine | M166T96 | 166.0184 | 96 | 12.69626 | C6H6N4S | 31820847.2 | 20771849.1 | | 0.038508 | 1.177987 | neg |
| Palmitoylethanolamide | M300T958 | 300.2887 | 957.8 | 3.250206 | C18H37NO2 | 121564713 | 87015655.8 | | 0.038798 | 1.185261 | pos |
| Riboflavin reduced | M378T453_1 | 378.1691 | 453.3 | 8.801659 | C17H22N4O6 | 32195137 | 11697961.7 | | 0.039254 | 1.29604 | pos |
| N-Acetylmethionine | M190T244 | 190.0547 | 243.8 | 1.978378 | C7H13NO3S | 29152375.6 | 9396843.42 | | 0.040147 | 1.185446 | neg |
| N-[(2S)-2-Amino-2-carboxyethyl]-L-glutamate | M235T93 | 235.093 | 93.2 | 2.22891 | C8H14N2O6 | 45391050.2 | 23389442.7 | | 0.040159 | 1.16681 | pos |
| (S)-Reticuline | M329T458_2 | 329.1489 | 458.1 | 1.37636 | C19H23NO4 | 4574251.14 | 2394921.15 | | 0.040371 | 1.190846 | pos |
| Butyryl-L-carnitine | M232T379 | 232.1547 | 378.9 | 0.002568 | C11H21NO4 | 1395084062 | 361269255 | | 0.041219 | 1.141645 | pos |
| N-Alpha-acetyllysine | M188T444 | 188.0704 | 443.8 | 1.774044 | C8H16N2O3 | 14681425.5 | 6535989.04 | | 0.041831 | 1.306116 | pos |
| 14alpha-Hydroxy-5beta-cholest-7-ene-3,6-dione | M414T712 | 414.3004 | 712.2 | 0.031478 | C27H42O3 | 3588684.13 | 1261958.44 | | 0.042695 | 1.298183 | pos |
| 8-Amino-7-oxononanoate | M170T491 | 170.1176 | 491.2 | 24.88867 | C9H17NO3 | 14395042.3 | 5019560.87 | | 0.043912 | 1.280164 | pos |
| Isoproterenol | M194T310 | 194.1176 | 309.6 | 21.81152 | C11H17NO3 | 114498237 | 85052800.7 | | 0.044038 | 1.162688 | pos |
| 3-Methyl-2-oxovaleric acid | M129T314 | 129.0568 | 313.8 | 8.337414 | C6H10O3 | 10518682.6 | 4371552.31 | | 0.044194 | 1.129615 | neg |
| Diethylstilbestrol | M268T756 | 268.1472 | 756.4 | 3.356365 | C18H20O2 | 1381823.98 | 280159.66 | | 0.045005 | 1.117537 | neg |
| Methylmalonic acid | M117T718 | 116.9295 | 718.3 | 18.24712 | C4H6O4 | 83919400.2 | 42914124.9 | | 0.046064 | 1.115705 | neg |
| Dodecanedioic acid | M229T508 | 229.1451 | 507.8 | 2.513691 | C12H22O4 | 3675282.9 | 2358819.53 | | 0.047043 | 1.106208 | neg |
| Adenine | M136T122 | 136.0608 | 122.3 | 6.934928 | C5H5N5 | 32436892 | 21359511.3 | | 0.047596 | 1.122554 | pos |
| 3-Methylthiopropionic acid | M119T401_2 | 118.9723 | 400.5 | 28.38888 | C4H8O2S | 119379.78 | 1644601.61 | | 2.8E-07 | 1.737579 | neg |
| 4-Guanidinobutanoic acid | M146T117_2 | 146.0812 | 117.1 | 0.05465 | C5H11N3O2 | 11716573.9 | 44193535.8 | | 5.05E-05 | 1.68316 | pos |
| Niacinamide | M123T125 | 123.0551 | 125.2 | 1.430254 | C6H6N2O | 2774385216 | 4741119250 | | 5.28E-05 | 1.665158 | pos |
| (R)-4-Hydroxymandelate | M169T146 | 169.0607 | 146.1 | 13.28484 | C8H8O4 | 9933353.12 | 29077988.2 | | 0.000159 | 1.64257 | pos |
| 2-Phenylacetamide | M134T333 | 134.0615 | 333.1 | 2.804691 | C8H9NO | 530468.76 | 2470095.17 | | 0.000178 | 1.630237 | neg |
| Hippuric acid | M178T333 | 178.0513 | 333.3 | 2.111751 | C9H9NO3 | 18677433.4 | 80990825.9 | | 0.000266 | 1.612716 | neg |
| IMP | M349T139 | 349.0564 | 138.7 | 3.701273 | C10H13N4O8P | 71267201.3 | 2058978661 | | 0.001395 | 1.533316 | pos |
| Salicyluric acid | M194T126 | 194.0499 | 125.8 | 20.48958 | C9H9NO4 | 29391847.5 | 87902322.5 | | 0.002555 | 1.470255 | neg |
| Tauropine | M198T113 | 198.0435 | 113.1 | 2.140948 | C5H11NO5S | 11256751.8 | 27505144 | | 0.003489 | 1.449872 | pos |
| Xanthylic acid | M363T72 | 363.035 | 72.2 | 0.760258 | C10H13N4O9P | 7928189.98 | 13354855.8 | | 0.004421 | 1.431923 | neg |
| O-Acetylcarnitine | M204T290 | 204.1235 | 290.2 | 0.246983 | C9H18NO4 | 11682971.8 | 56795407.3 | | 0.00473 | 1.413094 | pos |
| Pyridoxamine 5'-phosphate | M247T152 | 247.0499 | 151.5 | 3.917646 | C8H13N2O5P | 438685.31 | 1077723.53 | | 0.005748 | 1.39154 | neg |
| Aminocarb | M209T367 | 209.1287 | 367.4 | 1.071111 | C11H16N2O2 | 4933397.43 | 24881008.4 | | 0.009903 | 1.355048 | pos |
| LysoPA(16_0_0_0) | M409T802_1 | 409.2353 | 802.3 | 1.769153 | C19H39O7P | 26126800 | 37082493.7 | | 0.010763 | 1.330099 | neg |
| Linatine | M259T125_1 | 259.0932 | 125.1 | 1.764153 | C10H17N3O5 | 50090861.2 | 74279454.1 | | 0.010906 | 1.329444 | pos |
| Dulcin | M181T467 | 181.0973 | 466.9 | 0.684715 | C9H12N2O2 | 18244291.1 | 55997845.1 | | 0.012352 | 1.346337 | pos |
| 5-Hydroxyindoleacetic acid | M192T336 | 192.0655 | 336.5 | 0.124957 | C10H9NO3 | 10212234 | 41651801.8 | | 0.014765 | 1.316819 | pos |
| L-Malic acid | M133T140 | 133.0149 | 139.6 | 5.082137 | C4H6O5 | 7594622.87 | 23358734.9 | | 0.015424 | 1.304586 | neg |
| Palatinose | M323T122 | 323.1006 | 121.9 | 9.254084 | C12H22O11 | 3320953.87 | 14343728.7 | | 0.017257 | 1.302955 | neg |
| L-Kynurenine | M207T311 | 207.0784 | 310.6 | 4.230282 | C10H12N2O3 | 1977388.18 | 5974771.67 | | 0.020421 | 1.25317 | neg |
| D-Xylose | M149T81 | 149.0457 | 81.3 | 1.180846 | C5H10O5 | 13155171 | 19225144.2 | | 0.024584 | 1.239825 | neg |
| S-Lactoylglutathione | M378T125 | 378.0992 | 124.6 | 4.073978 | C13H21N3O8S | 10494059 | 27723375.5 | | 0.025964 | 1.329805 | neg |
| UMP | M325T115 | 325.0421 | 114.9 | 3.076525 | C9H13N2O9P | 7492668.47 | 27665330.3 | | 0.027681 | 1.320823 | pos |
| Vanillylmandelic acid | M181T552 | 181.0495 | 551.8 | 3.314011 | C9H10O5 | 1023342.95 | 3432798.38 | | 0.028963 | 1.345687 | pos |
| Pyrrolidonecarboxylic acid | M130T435 | 130.0512 | 435.5 | 9.996063 | C5H7NO3 | 31745838.1 | 66836035.5 | | 0.029153 | 1.211327 | pos |
| (2R)-2-Hydroxy-3-(phosphonatooxy)propanoate | M185T81 | 184.9862 | 80.6 | 2.702904 | C3H7O7P | 18457288.6 | 81934680.2 | | 0.034557 | 1.268646 | neg |
| 8,12-Diethyl-3-vinylbacteriochlorophyllide d | M570T772 | 570.2821 | 772.1 | 3.35216 | C34H34MgN4O3 | 22967331.9 | 51277161.9 | | 0.038128 | 1.167544 | pos |
| 2-Furoate | M111T109 | 111.0092 | 109 | 4.287933 | C5H4O3 | 3075282.17 | 6544182.98 | | 0.04113 | 1.152009 | neg |

Hits, the total number of substances in the target metabolic pathway; Up_hits, the number of up-regulated differential metabolites in the target metabolic pathway; Down_hits, the number of down-regulated differential metabolites in the target metabolic pathway.

**Table S4.** KEGG Pathway enrichment analysis of metabolites in soft palate tissue.

| Pathway_id | Pathway_name | Total | Hits | Up_hits | Down_hits | P | Impact |
| --- | --- | --- | --- | --- | --- | --- | --- |
| rno05230 | Central carbon metabolism in cancer | 37 | 8 | 6 | 2 | 0.000531 | 0.1698 |
| rno04024 | cAMP signaling pathway | 25 | 5 | 5 | 0 | 0.00868 | 0.1852 |
| rno00360 | Phenylalanine metabolism | 60 | 8 | 6 | 2 | 0.012462 | 0.119 |
| rno04917 | Prolactin signaling pathway | 11 | 3 | 3 | 0 | 0.017429 | 0.3158 |
| rno04080 | Neuroactive ligand-receptor interaction | 52 | 7 | 7 | 0 | 0.018092 | 0.1346 |
| rno00240 | Pyrimidine metabolism | 65 | 8 | 7 | 1 | 0.019659 | 0.2783 |
| rno00620 | Pyruvate metabolism | 31 | 5 | 3 | 2 | 0.021502 | 0.1088 |
| rno04721 | Synaptic vesicle cycle | 12 | 3 | 3 | 0 | 0.022352 | 0.25 |
| rno00410 | beta-Alanine metabolism | 32 | 5 | 5 | 0 | 0.024427 | 0.1034 |
| rno04742 | Taste transduction | 32 | 5 | 3 | 2 | 0.024427 | 0.1842 |
| rno02010 | ABC transporters | 138 | 13 | 12 | 1 | 0.028365 | 0.0942 |
| rno04922 | Glucagon signaling pathway | 26 | 4 | 2 | 2 | 0.045159 | 0.1364 |
| rno00310 | Lysine degradation | 50 | 6 | 6 | 0 | 0.045824 | 0.1234 |
| rno00190 | Oxidative phosphorylation | 16 | 3 | 3 | 0 | 0.048751 | 0.2 |
| rno05030 | Cocaine addiction | 7 | 2 | 2 | 0 | 0.048832 | 0.3636 |
| rno00350 | Tyrosine metabolism | 78 | 8 | 7 | 1 | 0.051265 | 0.152 |
| rno00250 | Alanine, aspartate and glutamate metabolism | 28 | 4 | 4 | 0 | 0.057036 | 0.1208 |
| rno05032 | Morphine addiction | 8 | 2 | 2 | 0 | 0.062892 | 0.2 |
| rno00760 | Nicotinate and nicotinamide metabolism | 55 | 6 | 5 | 1 | 0.067488 | 0.2279 |
| rno00380 | Tryptophan metabolism | 83 | 8 | 6 | 2 | 0.069179 | 0.1239 |
| rno04727 | GABAergic synapse | 9 | 2 | 2 | 0 | 0.07812 | 0.2353 |
| rno05031 | Amphetamine addiction | 9 | 2 | 2 | 0 | 0.07812 | 0.3077 |
| rno00020 | Citrate cycle (TCA cycle) | 20 | 3 | 2 | 1 | 0.085189 | 0.0778 |
| rno04217 | Necroptosis | 10 | 2 | 2 | 0 | 0.094356 | 0.1667 |
| rno05034 | Alcoholism | 10 | 2 | 2 | 0 | 0.094356 | 0.2857 |
| rno05012 | Parkinson disease | 21 | 3 | 3 | 0 | 0.095693 | 0.2121 |
| rno00590 | Arachidonic acid metabolism | 75 | 7 | 7 | 0 | 0.098372 | 0.0711 |
| rno04974 | Protein digestion and absorption | 47 | 5 | 5 | 0 | 0.099345 | 0.1064 |
| rno04672 | Intestinal immune network for IgA production | 2 | 1 | 1 | 0 | 0.102809 | 0.5 |
| rno05231 | Choline metabolism in cancer | 11 | 2 | 2 | 0 | 0.11145 | 0.191 |
| rno04020 | Calcium signaling pathway | 11 | 2 | 2 | 0 | 0.11145 | 0.2667 |
| rno00270 | Cysteine and methionine metabolism | 63 | 6 | 4 | 2 | 0.112641 | 0.141 |
| rno04714 | Thermogenesis | 23 | 3 | 3 | 0 | 0.118167 | 0.1026 |
| rno00260 | Glycine, serine and threonine metabolism | 50 | 5 | 4 | 1 | 0.121265 | 0.2019 |
| rno00230 | Purine metabolism | 95 | 8 | 6 | 2 | 0.126127 | 0.2269 |
| rno04728 | Dopaminergic synapse | 12 | 2 | 2 | 0 | 0.12927 | 0.2727 |
| rno05222 | Small cell lung cancer | 3 | 1 | 1 | 0 | 0.150201 | 0.3333 |
| rno05320 | Autoimmune thyroid disease | 3 | 1 | 1 | 0 | 0.150201 | 0.3333 |
| rno05410 | Hypertrophic cardiomyopathy | 3 | 1 | 1 | 0 | 0.150201 | 0.3333 |
| rno05211 | Renal cell carcinoma | 3 | 1 | 0 | 1 | 0.150201 | 0.4 |
| rno04726 | Serotonergic synapse | 42 | 4 | 3 | 1 | 0.178656 | 0.0968 |
| rno00650 | Butanoate metabolism | 42 | 4 | 4 | 0 | 0.178656 | 0.1618 |
| rno04071 | Sphingolipid signaling pathway | 15 | 2 | 2 | 0 | 0.185901 | 0.2069 |
| rno04978 | Mineral absorption | 29 | 3 | 3 | 0 | 0.195013 | 0.0857 |
| rno04142 | Lysosome | 4 | 1 | 1 | 0 | 0.195102 | 0.25 |
| rno04210 | Apoptosis | 4 | 1 | 1 | 0 | 0.195102 | 0.25 |
| rno05226 | Gastric cancer | 4 | 1 | 1 | 0 | 0.195102 | 0.25 |
| rno00770 | Pantothenate and CoA biosynthesis | 30 | 3 | 3 | 0 | 0.208855 | 0.0893 |
| rno04964 | Proximal tubule bicarbonate reclamation | 17 | 2 | 1 | 1 | 0.225297 | 0.1327 |
| rno00630 | Glyoxylate and dicarboxylate metabolism | 62 | 5 | 3 | 2 | 0.227465 | 0.0436 |
| rno00330 | Arginine and proline metabolism | 78 | 6 | 4 | 2 | 0.227746 | 0.1029 |
| rno03320 | PPAR signaling pathway | 5 | 1 | 1 | 0 | 0.237645 | 0.2 |
| rno00640 | Propanoate metabolism | 48 | 4 | 4 | 0 | 0.246047 | 0.1077 |
| rno04976 | Bile secretion | 97 | 7 | 7 | 0 | 0.249879 | 0.0642 |
| rno00920 | Sulfur metabolism | 33 | 3 | 2 | 1 | 0.251539 | 0.0545 |
| rno04723 | Retrograde endocannabinoid signaling | 19 | 2 | 2 | 0 | 0.265236 | 0.1053 |
| rno00400 | Phenylalanine, tyrosine and tryptophan biosynthesis | 34 | 3 | 3 | 0 | 0.266056 | 0.1053 |
| rno00072 | Synthesis and degradation of ketone bodies | 6 | 1 | 1 | 0 | 0.27795 | 0.1364 |
| rno04621 | NOD-like receptor signaling pathway | 6 | 1 | 1 | 0 | 0.27795 | 0.1667 |
| rno04916 | Melanogenesis | 6 | 1 | 1 | 0 | 0.27795 | 0.1667 |
| rno04930 | Type II diabetes mellitus | 6 | 1 | 1 | 0 | 0.27795 | 0.1667 |
| rno05223 | Non-small cell lung cancer | 6 | 1 | 1 | 0 | 0.27795 | 0.1667 |
| rno00740 | Riboflavin metabolism | 20 | 2 | 2 | 0 | 0.285242 | 0.2857 |
| rno00970 | Aminoacyl-tRNA biosynthesis | 52 | 4 | 4 | 0 | 0.293704 | 0.0804 |
| rno04920 | Adipocytokine signaling pathway | 7 | 1 | 1 | 0 | 0.316137 | 0.1429 |
| rno05033 | Nicotine addiction | 7 | 1 | 1 | 0 | 0.316137 | 0.1429 |
| rno04977 | Vitamin digestion and absorption | 39 | 3 | 1 | 2 | 0.339621 | 0.0612 |
| rno00290 | Valine, leucine and isoleucine biosynthesis | 23 | 2 | 2 | 0 | 0.344747 | 0.129 |
| rno04915 | Estrogen signaling pathway | 8 | 1 | 1 | 0 | 0.352315 | 0.125 |
| rno05143 | African trypanosomiasis | 8 | 1 | 0 | 1 | 0.352315 | 0.125 |
| rno04913 | Ovarian steroidogenesis | 24 | 2 | 2 | 0 | 0.364262 | 0.0682 |
| rno00600 | Sphingolipid metabolism | 25 | 2 | 2 | 0 | 0.383556 | 0.1096 |
| rno04929 | GnRH secretion | 9 | 1 | 1 | 0 | 0.386589 | 0.0909 |
| rno04979 | Cholesterol metabolism | 10 | 1 | 1 | 0 | 0.419059 | 0.0556 |
| rno04261 | Adrenergic signaling in cardiomyocytes | 10 | 1 | 1 | 0 | 0.419059 | 0.1 |
| rno05133 | Pertussis | 10 | 1 | 1 | 0 | 0.419059 | 0.1 |
| rno00780 | Biotin metabolism | 28 | 2 | 2 | 0 | 0.439815 | 0.0312 |
| rno04072 | Phospholipase D signaling pathway | 11 | 1 | 1 | 0 | 0.44982 | 0.0476 |
| rno04540 | Gap junction | 11 | 1 | 1 | 0 | 0.44982 | 0.0909 |
| rno04919 | Thyroid hormone signaling pathway | 11 | 1 | 1 | 0 | 0.44982 | 0.0909 |
| rno00750 | Vitamin B6 metabolism | 29 | 2 | 1 | 1 | 0.457948 | 0.1493 |
| rno04921 | Oxytocin signaling pathway | 12 | 1 | 1 | 0 | 0.478961 | 0.0833 |
| rno04911 | Insulin secretion | 12 | 1 | 1 | 0 | 0.478961 | 0.2 |
| rno00010 | Glycolysis / Gluconeogenesis | 31 | 2 | 1 | 1 | 0.493177 | 0.0332 |
| rno05200 | Pathways in cancer | 31 | 2 | 1 | 1 | 0.493177 | 0.0909 |
| rno00471 | D-Glutamine and D-glutamate metabolism | 13 | 1 | 0 | 1 | 0.506567 | 0.0952 |
| rno04611 | Platelet activation | 14 | 1 | 1 | 0 | 0.532718 | 0.1429 |
| rno04066 | HIF-1 signaling pathway | 15 | 1 | 1 | 0 | 0.55749 | 0.0526 |
| rno00030 | Pentose phosphate pathway | 35 | 2 | 1 | 1 | 0.559155 | 0.0462 |
| rno04750 | Inflammatory mediator regulation of TRP channels | 35 | 2 | 2 | 0 | 0.559155 | 0.0769 |
| rno04924 | Renin secretion | 17 | 1 | 1 | 0 | 0.603185 | 0.0741 |
| rno00480 | Glutathione metabolism | 38 | 2 | 2 | 0 | 0.60451 | 0.0664 |
| rno00280 | Valine, leucine and isoleucine degradation | 42 | 2 | 2 | 0 | 0.659397 | 0.0277 |
| rno04931 | Insulin resistance | 20 | 1 | 0 | 1 | 0.663078 | 0.0417 |
| rno04918 | Thyroid hormone synthesis | 21 | 1 | 1 | 0 | 0.680972 | 0.058 |
| rno00232 | Caffeine metabolism | 22 | 1 | 1 | 0 | 0.697921 | 0.0294 |
| rno00430 | Taurine and hypotaurine metabolism | 22 | 1 | 1 | 0 | 0.697921 | 0.0294 |
| rno04152 | AMPK signaling pathway | 22 | 1 | 1 | 0 | 0.697921 | 0.0333 |
| rno04925 | Aldosterone synthesis and secretion | 22 | 1 | 1 | 0 | 0.697921 | 0.125 |
| rno00220 | Arginine biosynthesis | 23 | 1 | 1 | 0 | 0.713975 | 0.0063 |
| rno00120 | Primary bile acid biosynthesis | 47 | 2 | 2 | 0 | 0.719272 | 0.0488 |
| rno00565 | Ether lipid metabolism | 25 | 1 | 1 | 0 | 0.74358 | 0.0241 |
| rno00830 | Retinol metabolism | 25 | 1 | 1 | 0 | 0.74358 | 0.0435 |
| rno00564 | Glycerophospholipid metabolism | 52 | 2 | 2 | 0 | 0.770088 | 0.0463 |
| rno00983 | Drug metabolism - other enzymes | 52 | 2 | 2 | 0 | 0.770088 | 0.0473 |
| rno00591 | Linoleic acid metabolism | 28 | 1 | 1 | 0 | 0.782372 | 0.0789 |
| rno04216 | Ferroptosis | 29 | 1 | 1 | 0 | 0.793958 | 0.0233 |
| rno04070 | Phosphatidylinositol signaling system | 29 | 1 | 1 | 0 | 0.793958 | 0.0351 |
| rno00040 | Pentose and glucuronate interconversions | 56 | 2 | 1 | 1 | 0.804819 | 0.0776 |
| rno00730 | Thiamine metabolism | 31 | 1 | 1 | 0 | 0.815323 | 0.0222 |
| rno05022 | Pathways of neurodegeneration - multiple diseases | 32 | 1 | 1 | 0 | 0.825164 | 0.0625 |
| rno00561 | Glycerolipid metabolism | 38 | 1 | 0 | 1 | 0.874171 | 0.0065 |
| rno00140 | Steroid hormone biosynthesis | 99 | 3 | 3 | 0 | 0.902921 | 0.0319 |
| rno00592 | alpha-Linolenic acid metabolism | 44 | 1 | 1 | 0 | 0.909498 | 0.0152 |
| rno00052 | Galactose metabolism | 46 | 1 | 1 | 0 | 0.918924 | 0.0079 |
| rno00562 | Inositol phosphate metabolism | 47 | 1 | 1 | 0 | 0.923264 | 0.0356 |
| rno00053 | Ascorbate and aldarate metabolism | 50 | 1 | 1 | 0 | 0.934947 | 0.0325 |
| rno00051 | Fructose and mannose metabolism | 54 | 1 | 1 | 0 | 0.947818 | 0.0317 |
| rno00130 | Ubiquinone and other terpenoid-quinone biosynthesis | 92 | 2 | 2 | 0 | 0.960201 | 0.0187 |
| rno00520 | Amino sugar and nucleotide sugar metabolism | 108 | 2 | 1 | 1 | 0.981282 | 0.0172 |
| rno01040 | Biosynthesis of unsaturated fatty acids | 74 | 1 | 1 | 0 | 0.982742 | 0.0104 |
| rno00860 | Porphyrin and chlorophyll metabolism | 142 | 1 | 0 | 1 | 0.99962 | 0.002 |

Hits, the total number of substances in the target metabolic pathway; Up_hits, the number of up-regulated differential metabolites in the target metabolic pathway; Down_hits, the number of down-regulated differential metabolites in the target metabolic pathway.
